# Artificial Intelligence Empowered New Materials: Discovery, Synthesis, Prediction to Validation

**DOI:** 10.1007/s40820-025-01945-4

**Published:** 2026-01-10

**Authors:** Ying Cao, Hong Fu, Jian Lu, Yuejiao Chen, Titao Jing, Xi Fan, Bingang Xu

**Affiliations:** 1 Nanotechnology Center, School of Fashion and Textiles, The Hong Kong Polytechnic University, Hong Kong, 999077 People’s Republic of China; 2 Department of Mathematics and Information Technology, The Education University of Hong Kong, Hong Kong, 999077 People’s Republic of China; 3 State Key Laboratory for Powder Metallurgy, Central South University, Changsha, 410083 People’s Republic of China; 4 School of Chemical Engineering and Technology, Sun Yat-Sen University, Zhuhai, 519082 People’s Republic of China; 5 Ningbo Institute of Materials Technology and Engineering, Chinese Academy of Sciences, Ningbo, 315201 People’s Republic of China

**Keywords:** Artificial intelligence, Material discovery and cognition, Design tactics, Review and perspective

## Abstract

Recent years have witnessed the significant breakthrough in the field of new materials discovery brought about by the artificial intelligence (AI). AI has successfully been applied for predicting the formability, revealing the properties, and guiding the experimental synthesis of materials. Rapid progress has been made in the integration of increasing database and improved computing power. Though some reviews present the development from their unique aspects, reviews from the view of how AI empowered both discovery of new materials and cognition of existing materials that covers the completed contents with two synergistical aspects are few. Here, the newest development is systematically reviewed in the field of AI empowered materials, reflecting advanced design of the intelligent systems for discovery, synthesis, prediction and validation of materials. First, background and mechanisms are briefed, after which the design for the AI systems with data, machine learning and automated laboratory included is illustrated. Next, strategies are summarized to obtain the AI systems for materials with improved performance which comprehensively cover the aspects from the in-depth cognizance of existing material and the rapid discovery of new materials, and then, the design thought for future AI systems in material science is pointed out. Finally, some perspectives are put forward.

## Highlights

- A comprehensive review focused on the recent advancement of artificial intelligence (AI) powered materials research from various aspects, including material discovery, synthesis, prediction and validation, is presented.
- The design strategies for the enhanced performance of AI for materials can be implemented from various procedures for cognizance of existing materials and discovery of novel materials with the data processing, algorithm design and automated laboratory construction included.
- A broad outlook on the future considerations of the AI systems for material is proposed.

## Graphical abstract

**Figure:**
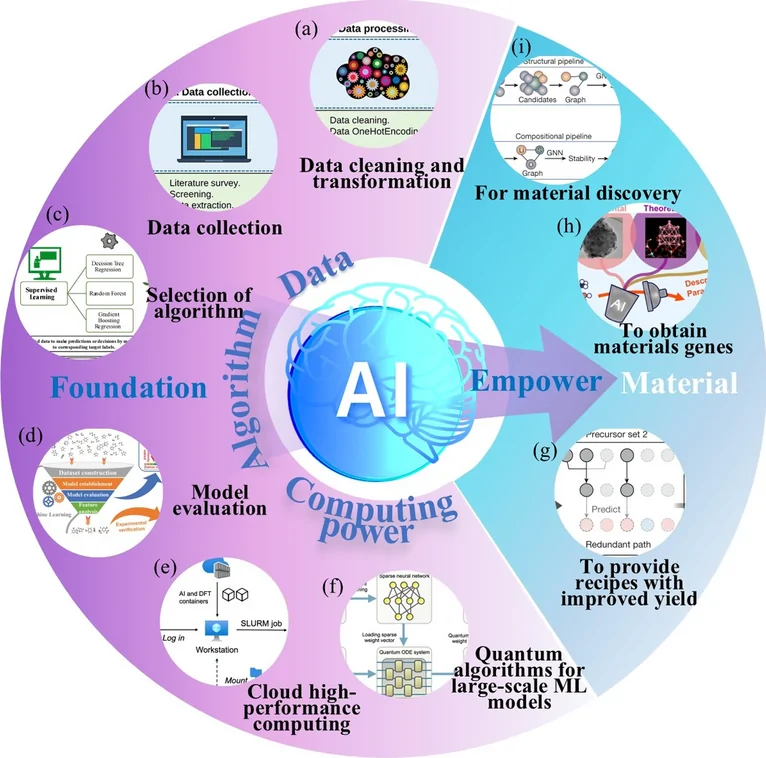

## Introduction

**Fig. 1:**
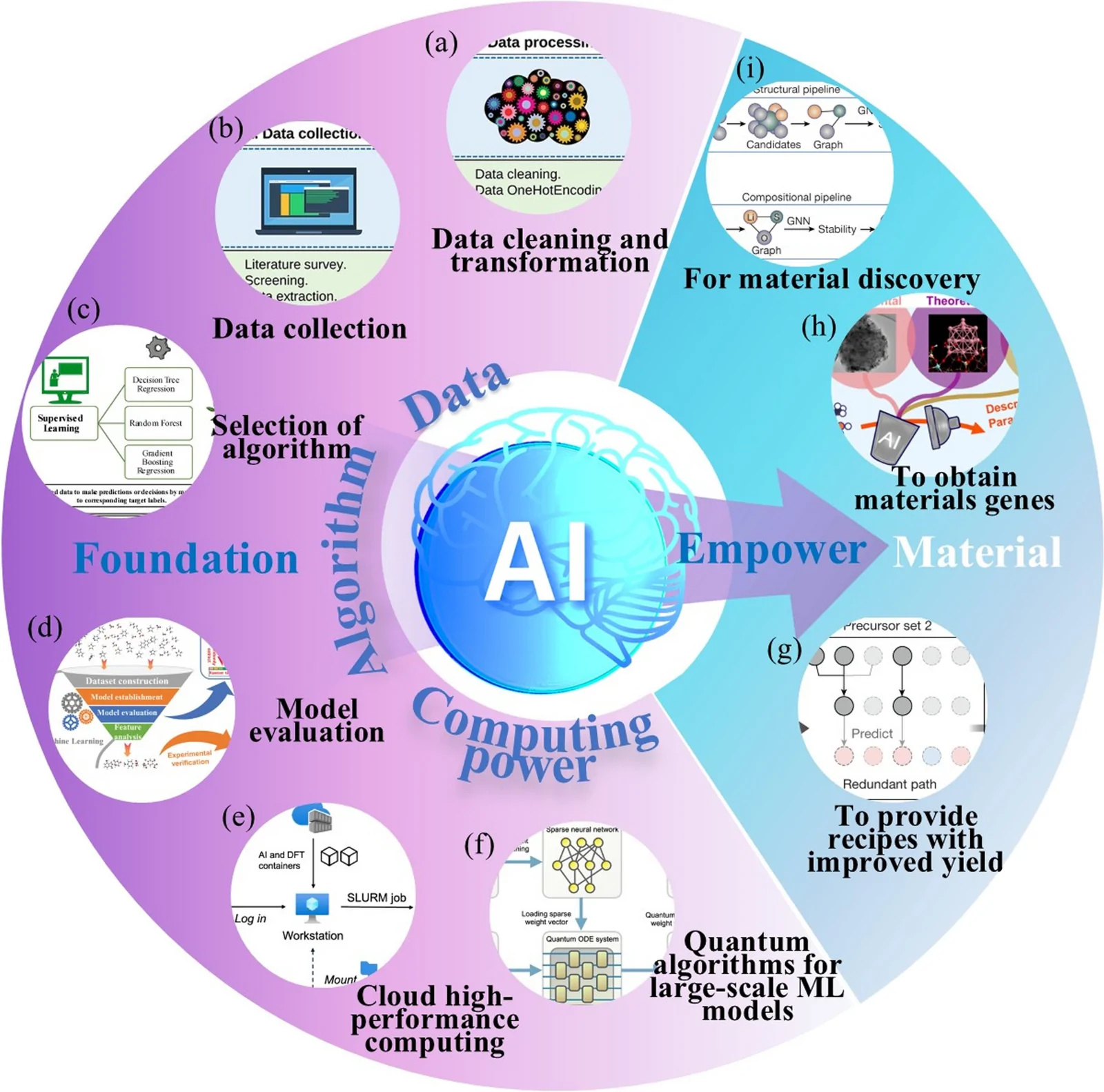
Overview of AI for materials with the data, algorithm, and computing power as fundamentals to support materials discovery and cognizance. **a** and **b** Reproduced with permission from Ref. [96]. Copyright 2022, Elsevier. **c** Reproduced under the terms of the CC-BY license [97]. Copyright 2024, The Authors, published by Wiley. **d** Reproduced with permission from Ref. [98]. Copyright 2024, Wiley–VCH GmbH. **e** Reproduced with permission from Ref. [99]. Copyright 2024, American Chemical Society. **f** Reproduced under the terms of the CC-BY license [100]. Copyright 2024, The Authors, published by Nature. **g** Reproduced under the terms of the CC-BY license [101]. Copyright 2023, The Authors, published by Nature. **h** Reproduced under the terms of the CC-BY license [91]. Copyright 2024, The Authors, published by American Chemical Society. **i** Reproduced under the terms of the CC-BY license [51]. Copyright 2023, The Authors, published by Nature

The discovery and application of advanced materials and devices have promoted humans to combat the major global challenges [1–9]. Artificial intelligence (AI) has proved to be powerful tools for new material discovery [10–18], device performance prediction [19–24], and system performance improvements [25–32], and the emergent predictive capability has been verified with the assistant of increasing data, advanced algorithms and improved computing power [33–41] (Fig. 1). In particular, many novel information processing systems are developed, which will facilitate the progress made in the material science [42–46]. At the meantime, the rapid progress in the field of functional materials and devices has proposed high demand for AI [8, 47–50]. Novel methods for generating diverse candidate structures can be created, which can improve the efficiency of material discovery to a large extend [51, 52]. A large number of novel structures can be discovered by AI, many of which are beyond what human intuition can reach. Furthermore, as to the cognition of the existing materials, it is possible for AI to map the relationships between their structures and properties so as to make the prediction for previously uncharacterized properties [53–59] and device performances [60–72]. AI can also meet the challenge in illustrating the relationship between the physical properties of the stimuli in the external environment and their perceptual signals [73]. AI which can overcome the shortcomings of traditional trial-and-error method in material discovery and cognition has found its wide applications in many advanced functional materials [74–86], like two-dimensional perovskites [87], multicomponent oxides [88], nanomaterial [89], and silicon-oxygen compounds [90], which has prompted the development of many domains, such as information processing, clean energy harvesting, and catalysis discovery [51, 91–95].

The experimental synthesis of materials is also facilitated greatly by AI since the data-driven techniques, especially machine learning (ML), are managed to find the structure-property relationships of the materials, indicating the types of materials that are more feasible to be prepared, which used to be very difficult and time-consuming for humans to find the suitable methods and prepare new materials [87]. Moreover, efficient synthesis recipes can also be offered with the assistance from AI, which can simplify the manufacturing of complex materials and accelerate the synthesis of theoretically predicted materials to a large extend [88]. High throughput and reproducibility can be realized at the same time by the robotic laboratories, making the exploration on the large-scale hypotheses to be rapid and reproducible [88].

**Fig. 2:**
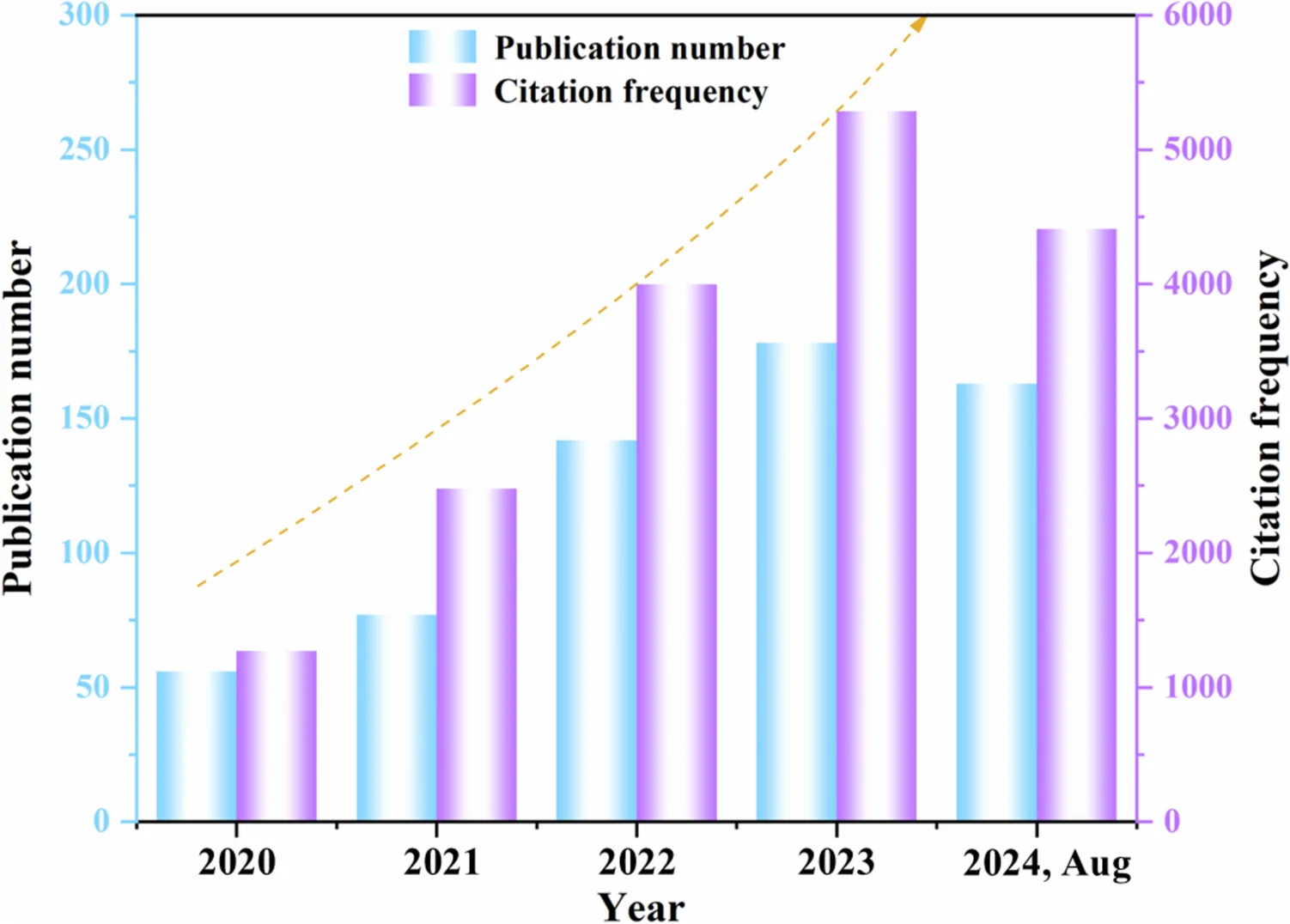
Publication number and citation frequency of the work focused on the artificial intelligence empowered materials discovery and prediction during the recent five years

**Fig. 3:**
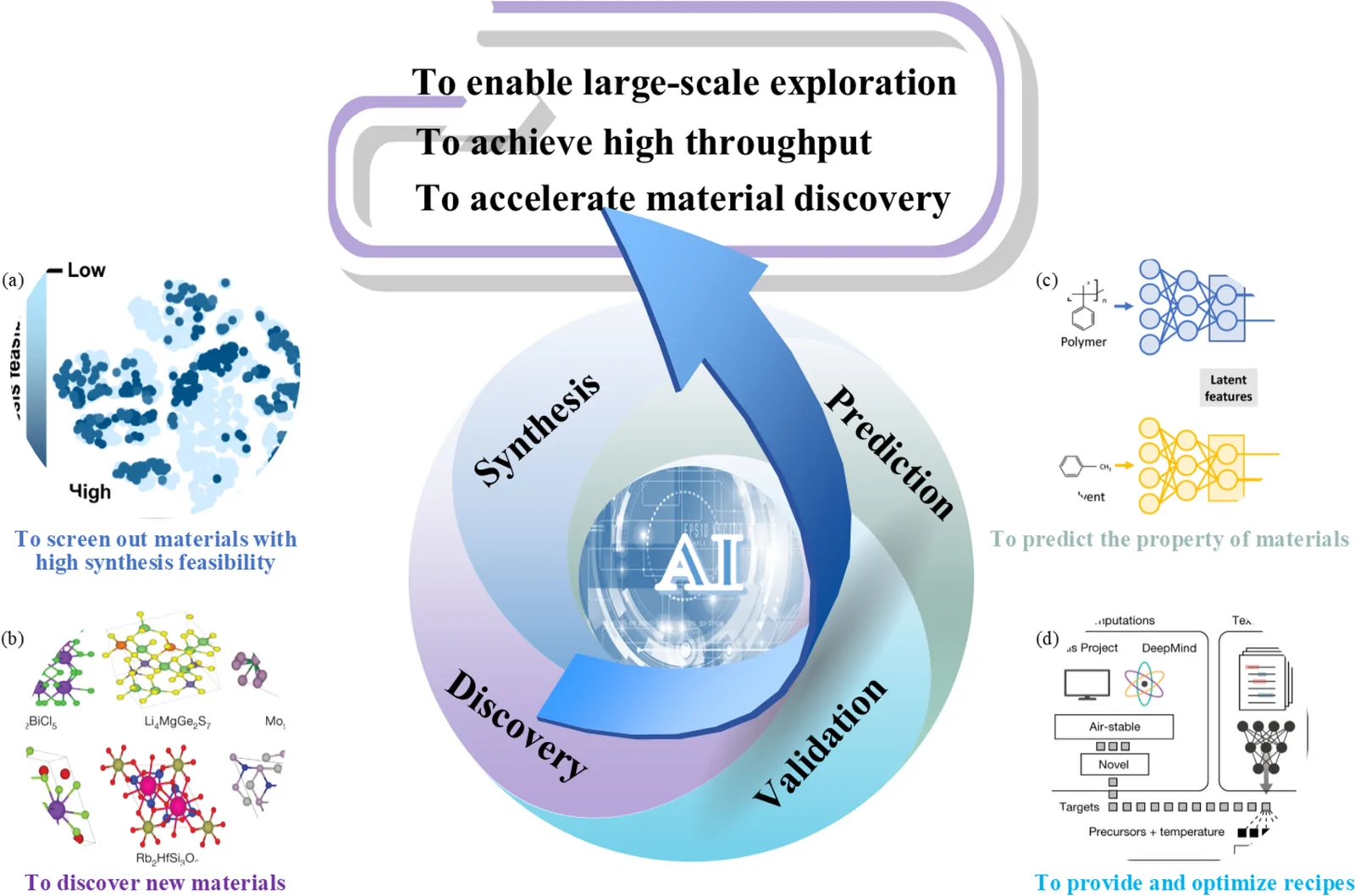
Tactics for the AI empowered material synthesis, discovery, prediction, and validation. **a** Reproduced under the terms of the CC-BY license. [87] Copyright 2024, The Authors, published by Nature. **b** Reproduced under the terms of the CC-BY license. [51] Copyright 2023, The Authors, published by Nature. **c** Reproduced under the terms of the CC-BY license. [108] Copyright 2023, The Authors, published by American Chemical Society. **d** Reproduced under the terms of the CC-BY license. [101] Copyright 2023, The Authors, published by Nature

Recent years have witnessed a rapid development of the AI for materials science. The discovery of 2.2 million structures below the current convex hull has been realized with the efficiency of materials discovery promoted by an order of magnitude, among which many have been beyond the previous human chemical intuition [51]. A principal odor map has been developed which can make odor quality prediction for previously uncharacterized odorants [53]. The accurate and fast structure-adsorption prediction has been made by DeepSorption, a spatial atom interaction learning network [102]. The structural information has been provided for the disordered silicon at very-high pressure of up to 20 GPa via atomistic ML models, offering the predications for the material systems even under experimentally challenging conditions [103]. As to the AI-assisted material synthesis, a universal framework has been developed for the preparation of two-dimensional perovskites with the ability of increasing the success rate of the synthesis feasibility by a factor of four compared to the traditional methods, which can be used in the typical laboratory [87]. It is noticeable that an autonomous laboratory has been successfully developed in order to achieve the accelerated synthesis of novel materials, which was managed to realize 41 novel compounds from a set of 58 targets under continuous operation of over17 days [101]. As a result, many original works of high quality have been published with the citation frequency growing sharply over time (Fig. 2). Tactics have been developed for the AI empowered materials from many aspects, including synthesis, discovery, prediction, and variation, to realize the large-scale exploration, high throughput, and accelerated material discovery, which is demonstrated in Fig. 3. Several reviews relevant to the AI for material science are reported, and each of them has its own emphasis, with how AI promotes the membrane design [104], catalyst exploration [105], and development of other functional materials [106] included. Besides, other reviews provide us with the inspirations from other useful aspects, like the importance of interpretable ML for materials [107]. However, reviews from the view of how AI empowered both discovery of new materials and cognition of existing materials that covers the completed contents with these two synergistical aspects of cognition and discovery are few.

To be specific, the basic background of AI systems powered material research was introduced first, and then, the latest development in regard to the data collection and processing, the algorithm selection, and the automated laboratory design for the AI systems applied in material science were demonstrated. Some important factors which should be under consideration when designing the advanced AI systems were discussed, including the strategies of how to obtain the systems with enhanced performance, the features of the future AI systems for materials, and so on. Last but not least, some ideas with respect to the outlook of AI for materials were proposed.

## Mechanism of AI for Cognizance of Existing Materials and Discovery of Novel Materials

**Fig. 4:**
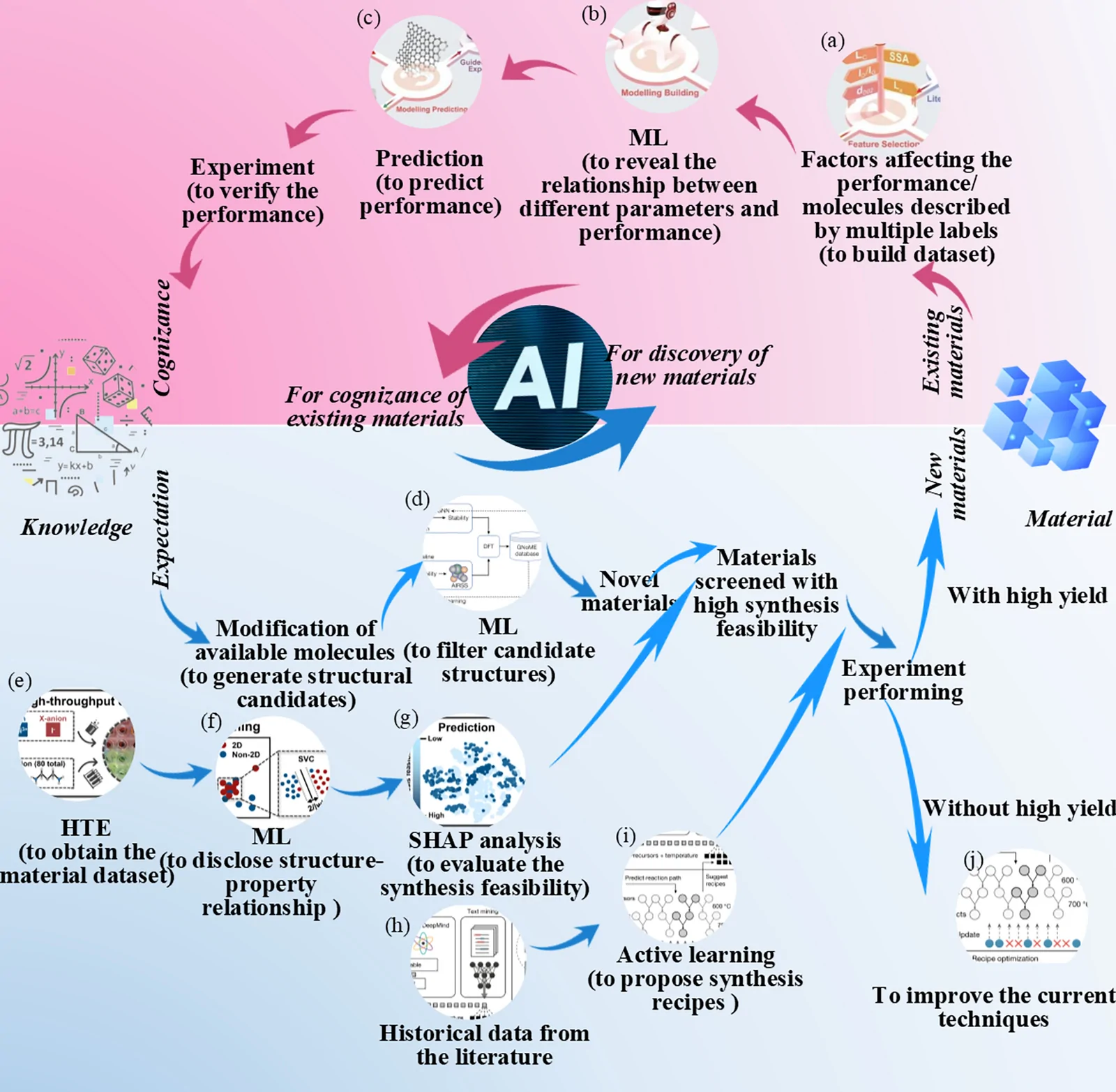
Schematic illustration for the mechanism of AI empowered material discovery and cognizance. **a**–**c** Reproduced with permission from Ref. [114]. Copyright 2023, The Royal Society of Chemistry. **d** Reproduced under the terms of the CC-BY license. [51] Copyright 2023, The Authors, published by Nature. **e**–**g** Reproduced under the terms of the CC-BY license. [87] Copyright 2024, The Authors, published by Nature. **h**–**j** Reproduced under the terms of the CC-BY license. [101] Copyright 2023, The Authors, published by Nature

Computing power plays a fundamental role in AI systems for materials, and data and algorithm are also of great importance in these systems [33, 109]. The improved computing power is managed to unlock modeling capabilities, which is beneficial for highly accurate and robust learning [51]. First-principles calculations based on density functional theory (DFT) have been made use of by computational approaches championed by the Materials Project (MP) [110], the Open Quantum Materials Database (OQMD) [111], NOvel MAterials Discovery (NOMAD) [112], and Automatic FLOW for materials discovery (AFLOWLIB) [113]. As shown in Fig. 4, the mechanism related to how AI empowers material research can be mainly illustrated from the aspects of the existing material cognizance and the novel materials discovery.

AI makes contribution to map molecular structures to their properties in regard to cognition of the existing materials [115], so that the relationships can be got and prediction of the properties for previously uncharacterized materials can be made [53]. It is worthwhile mentioning that efforts have been made to deal with the situation where the structurally similar pair is not the perceptually similar pair, and the predictive modeling in diverse perceptual aspects has been realized by neural networks [116]. As a specific type of graph neural network (GNN), the message passing neural network (MPNN) can be used to map chemical structures to percepts. Each molecule was described as a graph with each atom and bonds represented by a series of characters in details, after which the fragment weights can be optimized. A reference dataset of many molecules described by multiple corresponding property labels is needed to be curated (Fig. 4a). The models are then to be trained with their parameters being optimized to generate the maps (Fig. 4b). The reliability of the model in describing the properties can then be verified by experiments to justify whether a generalized description of structure-property relationships can be obtained (Fig. 4c), and the results can also be compared with that of the conventional structure-based maps to verify its efficiency.

In addition to the cognizance of the existing materials, AI also plays a vital role in the discovery of novel material [117–121]. Researchers are capable of conducting searches by substituting similar ions and enumerating prototypes, and endeavors have been made to improve the search efficiency of these approaches. In order to obtain more diverse candidates, neural networks can be applied to guide the searches [51]. It is proposed that a broader exploration can be made by neural networks while maintaining the efficiency. To be specific, structural candidates can be obtained by modifications of available materials, and methods have been built to enable incomplete replacements, so that the set of substitutions can be augmented largely. DFT, which is an important method for calculating material properties in materials science, plays a fundamental role of bridging between the microscopic electronic structure and the macroscopic properties of materials. As for the structural candidates, the energy and other key properties of materials are calculated through DFT to verify the accuracy of the model’s prediction. The new data obtained from DFT calculations can be added to the training set to train more powerful and robust models in the next round of active learning. Large scale of new materials can also be identified by means of high-throughput computation [101]. For instance, large-scale ab initio phase-stability data can be gathered from the MP and Google DeepMind [101].

AI is indispensable for the accelerated realization of new materials and the optimization of their design rules [91, 122–127]. After the identification of new materials, ML can then be applied to screen the novel materials with excellent performance and high synthesis feasibility (Fig. 4d-g). The critical physicochemical parameters related to the measured performance can be identified as materials genes among many candidate parameters obtained from experiments and initio simulations. By means of using a ML model, novel materials with high performance are able to be suggested. Various reaction conditions, including precursors, intermediate products, additives, solvents, and temperature, should be taken into considerations for materials synthesis, which usually consumes a lot of time for the experts [128]. The data-driven techniques are now used for screening out materials with high synthesis feasibilities by means of finding out the structure-property relationships, making it possible for the experimental realization of computational predictions [87].

The autonomous laboratory can be introduced to bridge the computational screening and experimental realization [101]. By the combined usage of computations, historical data, and ML, the plan and interpreting of the experimental outcomes can be made (Fig. 4h). ML model is able to provide the initial synthesis recipes for the proposed compounds, which can promote the material preparation (Fig. 4i). To be specific, the initial synthesis recipes can be obtained by the natural-language models learning to evaluate target ‘similarity’ via natural-language processing of a large database from the literature, which is similar to how a human make an attempt to begin initial synthesis according to known related materials [129]. It is worthwhile mentioning that analysis of the failed syntheses makes sense to offer direct guidance to improve materials screening and synthesis design [101], which is illustrated in Fig. 4j. Experiments will continue by taking advantages of autonomous reaction route optimization and solid-state synthesis, which is an active learning algorithm integrating ab initio computed reaction energies with observed outcomes once the yield does not achieve the expectation [130]. Experiments can be conducted by robotics. It is verified that autonomous workflows based on liquid handling can be demonstrated in organic chemistry [131–134], and recently, it is also possible for A-Lab to handle and characterize solid inorganic powders which used to be a challenge [101].

## Design of the Intelligent Systems for New Materials

### Data Collection

**Fig. 5:**
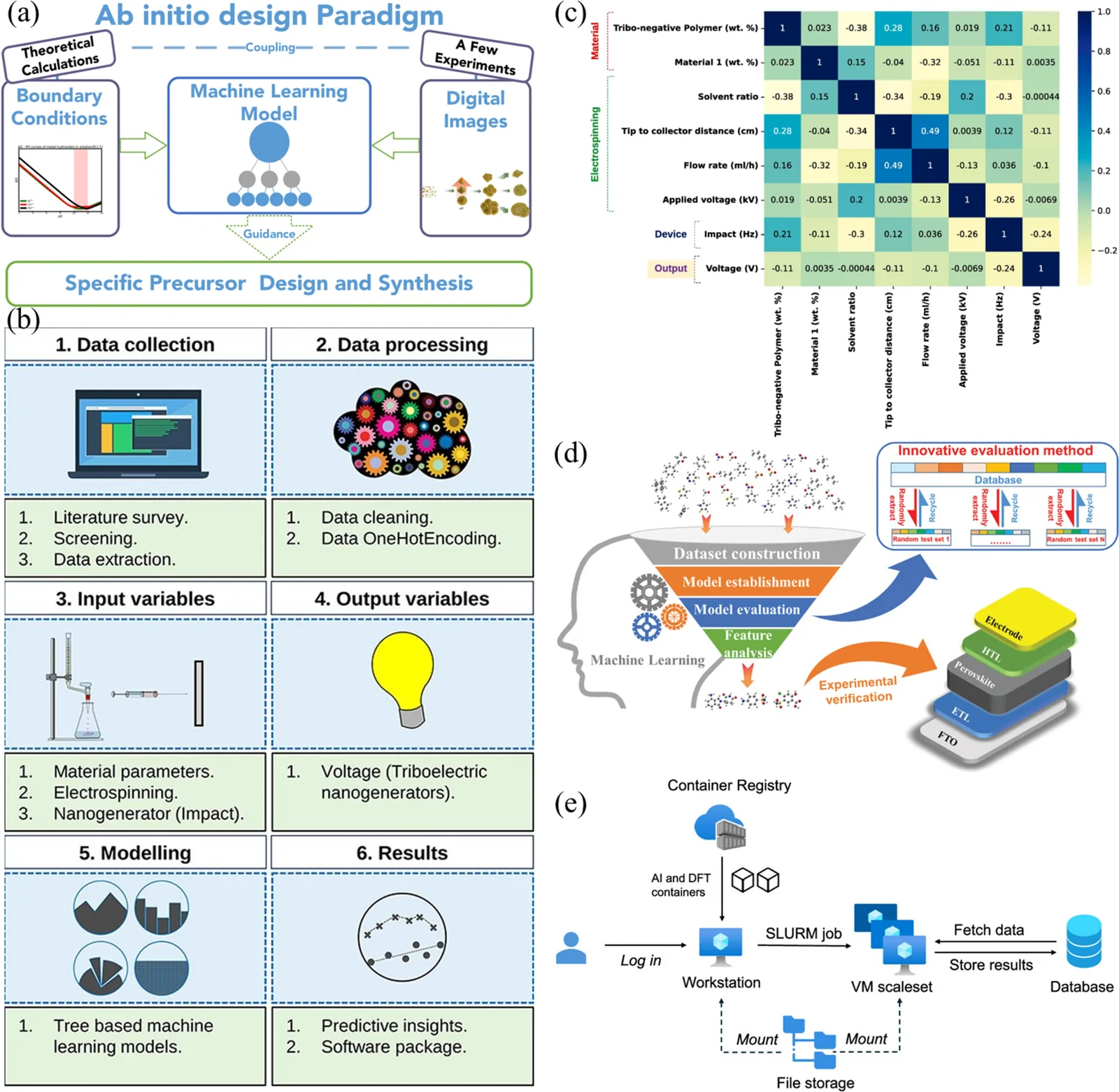
**a** Schematic illustration of the design synthesis paradigm incorporated with ML, which indicated the resources for the database. Reproduced under the terms of the CC-BY license [97]. Copyright 2024, The Authors, published by Wiley.** b** Schematics of the procedures for modeling the TENG ML models with data cleaning conducted. **c** Pearson correlation coefficient between input and output. Reproduced with permission from Ref. [96] Copyright 2022, Elsevier.** d** Schematic illustration of the ML process with the novel evaluation method to deal with the occasion where the available dataset was limited. Reproduced with permission from Ref. [98] Copyright 2024, Wiley–VCH GmbH. **e** Schematic illustration of the cloud environment for materials discovery workloads. Reproduced with permission from Ref. [99] Copyright 2024, American Chemical Society

Material data paly fundamental and important roles in the intelligent systems [135]. To be specific, the experimental synthesis data provided by studies, the first-principles calculations, and laboratory experience can be served as resources for the database [87]. For example, a design synthesis paradigm incorporated with ML was developed for Ni-rich cathode material, in which the boundary conditions for various reactions of precursors were provided by thermal/kinetic simulations, and a digital image dataset was constructed by some necessary experiments [18] (Fig. 5a).

Abundant datasets with balanced data to overcome the problems of overfitting, underfitting, and limited extrapolating abilities of ML are expected to be provided [136, 137]. The data processing that includes data cleaning and data transformation can be carried out to make sure that the collected data are integrated [96]. For example, in an attempt to develop the predictive models for real-time voltage output of triboelectric nanogenerator (TENG), data cleaning was conducted to eliminate incomplete or inconsistent data, leading to a refined dataset with 279 reliable data points, which guaranteed the quality and consistency of the dataset (Fig. 5b). Pearson correlation coefficient which revealed the linear relationship between the two variables was demonstrated in Fig. 5c. Particularly, a negative correlation demonstrated that when the values of these parameters increased, there was a high probability for the output voltage to decrease, and then, the specific mechanisms underlying these relationships could be further investigated.

Efforts have been made to meet the challenge of limited available dataset for model evaluation [98]. For instance, a novel evaluation method was developed to screen small molecules served as passivation materials for perovskite solar cell when the available dataset was limited (Fig. 5d). Particularly, 20% of test data were randomly extracted, while the remaining parts were used as the training data, followed by calculating model accuracy which was a critical criterion for the evaluation of classification model. The extracted data were then reintegrated into the established dataset, and another 20% of the data was randomly selected as a test set, the process of which was repeated 100 times. The final model accuracy was then obtained as the averaging of the accuracy values from these 100 calculations.

High-performance computing (HPC) is another strong support for the accelerated and large-scale material discovery and cognizance [99]. Endeavors have been made to put forward the strategies which can offer large-scale computational resources for the screening and experimental validation. It is pointed out that cloud HPC can meet this challenge which has been verified to be managed to train and host large-scale AI models like GPT-4 asking for a massive number of graphical processing units (GPUs), and therefore, it is promising to be applied for material research with an increasing number of material candidates to be evaluated computationally. One case in point was that ML and cloud HPC were combined, and the schematic illustration of the cloud environment for materials discovery is shown in Fig. 5e. Particularly, the ML models and DFT code were built into Docker container images. When operated, a workstation virtual machine (VM) fetched the container images to NetApp Files storage mounted to the workstation and job queues or VM scale sets. The computational jobs were submitted to the VM scale sets via the SLURM job scheduler. Data and metadata were kept in a searchable database. It was proved that the system was managed to quickly navigate through more than 32 million candidates as well as predict around half a million potentially stable materials.

Another issue that cannot be ignored is that the training data used in many studies is often biased toward successful cases reported in the literature or databases, which will lead to the inconsistence between the data distribution and the real-world distribution. This imbalance can leave an impact on the generalization ability and robustness of the models. Some strategies can be taken advantages of to address these issues. For instance, negative sample construction is one of the most fundamental strategies. Besides, active learning enables the model to actively select the samples that most need labeling, prioritizing the supplementation of negative or marginal samples which are most crucial for the model’s improvement. In addition, the multi-source data integration is another critical method for solving this problem. By integrating data from different sources and of different types, the sample distribution is enriched, thereby reducing the bias.

### Machine Learning Algorithms

The development of data-driven techniques has significantly revolutionized the new material discovery, which is able to provide physical insights from the existing data in depth [87]. ML has developed rapidly to meet the multidimensional challenges in the material field [138–146]. ML can be used to reveal the structure-property relationship hidden behind a large number of experiments. Materials with high synthesis feasibility can be screened out with the assistance from ML, accelerating material synthesis even with limited experimental support [87]. Rapid predictions for structures and properties can be made by ML even for new materials.

The effective transformation of experimental data into model-ready input features plays fundamental and important role in building intelligent models, and some explorations have been made about how to realize the effective transformation. In some cases, the differential features, rather than the original curves or data, are focused. Besides, instead of processing data with a single branch, the integration of two learning perspectives is carried out, making it possible for the models to learn from two dimensions. Additionally, designs can be conducted by making the input features highly correlated with the prediction target, reducing the learning burden of the model, and therefore, the prediction efficiency can be improved. One case in point was that a deep learning (DL) framework designed for the prediction of battery lifetime was developed by introducing an inter-cell learning mechanism to make prediction of the lifetime differences between two battery cells with the aim to represent the connections between cells cycled under different conditions [147]. In addition, the cycle-level features were fed into two separate branches, which contains the intra-cell difference curves and the inter-cell difference curves. Moreover, the correlations between the constructed features and the prediction targets for both intra-cell and inter-cell learning were investigated, and it was verified that a simple feature computed on inter-cell difference curves was managed to differentiate lifetime differences, even with a reference cell from a different battery chemistry, indicating its direct relationship between the constructed features and the prediction targets.

The appropriate selection and adaptation of models are imperative to develop the AI systems for the material science. It is worthwhile mentioning that the features of the model should be matched with the task requirements and data characteristics. To be specific, the complexity of the data should be evaluated, and then, corresponding models which are suitable for the tasks can be utilized. For instance, some basic linear models relying such as the ‘Var.’ and ‘Dis.’ can show commendable performance on the initial datasets, while they are not qualified for some complex datasets. Besides, some models relying on handcrafted features can be suitable for the task scenarios with stable data distribution, while for the tasks with diverse scenarios, models with the features of automatic learning should be given priority. Additionally, diverse and highly challenging test sets can be used to evaluate whether the models are appropriate.

**Fig. 6:**
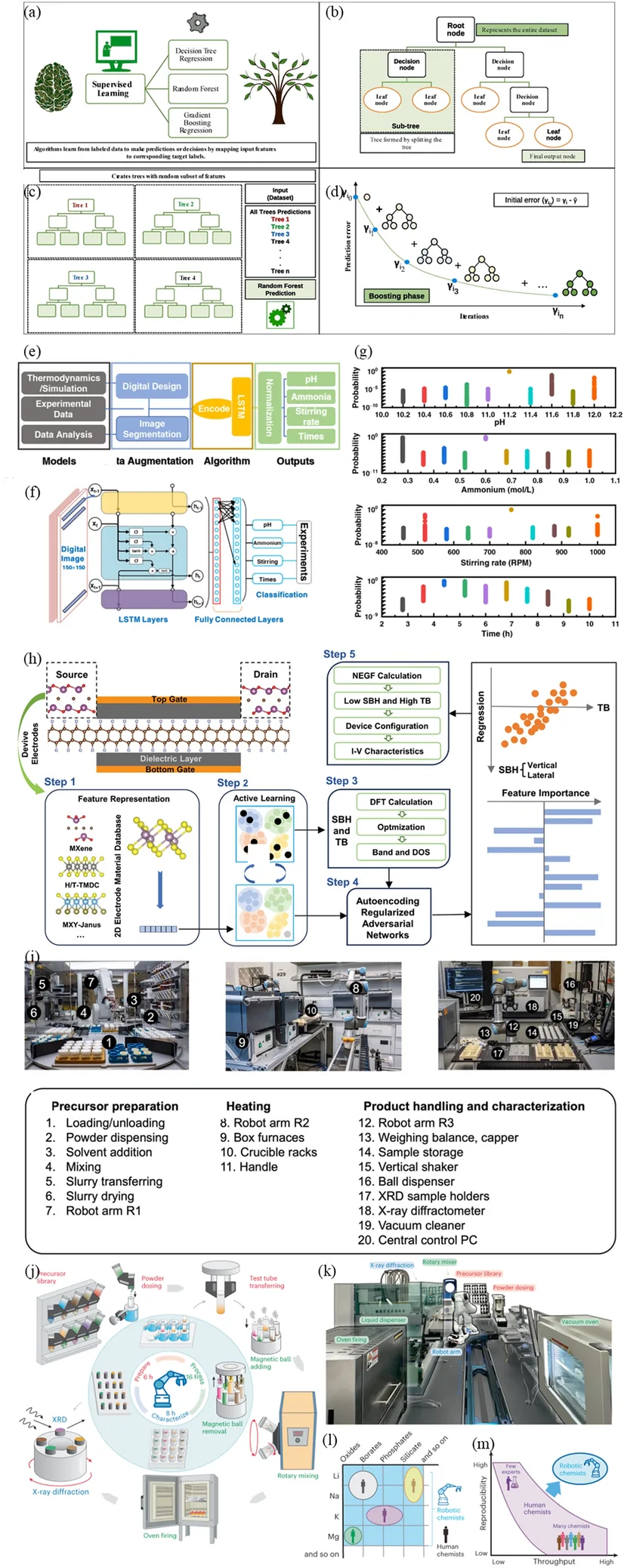
**a** Different algorithms with their own characteristics for the predictive TENG framework. Schematic of **b** DTR, **c** RF, and **d** GBR. Reproduced with permission from Ref. [96] Copyright 2022, Elsevier. **e** Computational flow of the ML for precursor design. **f** Model based on PMD-LSTM algorithm. **g** ML-assisted design for 3 μm precursors. Reproduced under the terms of the CC-BY license [97]. Copyright 2024, The Authors, published by Wiley. **h** Workflow of the efficient screening of 2DEMs. Reproduced with permission from Ref. [157] Copyright 2024, Wiley. **i** Details of the A-Lab. Reproduced under the terms of the CC-BY license. [101] Copyright 2023, The Authors, published by Nature. **j** Schematic illustration of the robotic inorganic materials synthesis, and **k** photograph of the laboratory. **l** Illustration for robotic chemists enabled large-scale exploration compared with human chemists. **m** Illustration for robotic chemists enabled both the high reproducibility and throughput. Reproduced under the terms of the CC-BY license. [88] Copyright 2024, The Authors, published by Nature

ML algorithms are selected according to classification accuracy, simplicity, computation efficiency, and so on [148–151]. Different ML algorithms have their own characteristics. For modeling with small dataset, support vector machine, linear regression, and gradient boosting are usually suitable [152, 153]. For example, in an attempt to improve the output performance of polyvinylidene fluoride (PVDF) nanogenerators, three decision tree ML models, including decision tree regression (DTR), random forest (RF), and gradient boosting regression (GBR), were chosen to develop the predictive models [96], which is illustrated in Fig. 6a. To be specific, DTR was extensively used for regression tasks, for which a binary tree structure was constructed by recursively splitting the TENG data based on the feature values (Fig. 6b). DTR was featured with its interpretability since the tree structure provided a clear visualization of the decision-making process. As shown in Fig. 6c, the RF combined multiple decision trees in order to enhance prediction accuracy, which was able to capture complex relationships between the input and output. As to GBR, decision trees were built sequentially with the subsequent tree reducing the errors made by the previous trees. The predictions of multiple weak models were incorporated to generate a strong model [154–156] (Fig. 6d). Another case in point was that the LSTM algorithm was applied in a design synthesis paradigm assisted with ML for Ni-rich cathode material, since the augmented datasets were still tiny [97] (Fig. 6e). It was proposed that the LSTM unit possessed its own advantages over RNN and CNN in the aspects of dealing with small sample data (Fig. 6f). ML-assisted design for 3 μm precursors is illustrated in Fig. 6g.

Recently, endeavors have also been made to develop atomic-scale models of complex materials [158, 159]. Large datasets and ML have emerged, which are suitable for these more complex systems [160]. One case in point was that active machine learning was applied to offer a unified computational description of the silicon-oxygen systems, which were among the most important materials with complexity [90].

Various approaches have been used to deal with small dataset limitations, such as transfer learning [161], autoencoders [162], and active learning [163, 164]. These methods can address the issues including noise, data imputation, and some other problems. Active learning strategies have been used more frequently for the classification scenarios compared to rigorous regression predictions. Recently, ML method has been adopted as the core component to screen low-contact electrode when limited data are available [157]. The detailed workflow is shown in Fig. 6h. To be specific, 2D electrode materials (2DEMs) were selected as numerical vectors using feature descriptors for the reason that it was verified to mitigate the Fermi level pinning (FLP) effect and maintain excellent gate control in low-dimensional devices (Step 1). The feature distribution was deemed as a baseline for active learning, and the representative data points were collected iteratively. The consistency between the feature distribution of the training subset and overall sample features was made use of as the active learning evaluation function (Step 2). An autoencoding regularized adversarial neural network (ARANet) to perform model training in a scenario with a limited contact-property dataset generated through DFT calculations was developed (Step 3 and 4). Moreover, a novel feature-adaptive variational active learning (FAVAL) algorithm was introduced to work with ARANet, obtaining a valuable training subset. It was worthwhile mentioning that the jointly trained FAVAL and ARANet schemes outperformed typical small-data models using the same training datasets. Preliminarily screened materials could be accomplished (Step 5). It was noticeable that this scheme showed exceptional performance when trained with only 15% of the total data points.

The applicability of ML models in dealing with small-sample data and complex material systems and the generalization ability of the models are all important aspects for the AI empowered materials systems. Some novel approaches have been put forward to deal with these problems. For instance, some new mechanisms have been adopted when tailoring the frameworks. It was pointed out that many models for the battery lifetime prediction were developed and validated only across a restricted set of aging conditions, and therefore, efforts should be made to improve their extensive applicability. In contrast to many traditional models that were mainly focused on intra-cell learning by means of capturing early variations of a single cell to implement the prediction of its long-term lifetime, a framework was proposed to integrate inter-cell learning [147]. It was worthwhile mentioning that the stability of lifetime predictions for a target cell under varied aging conditions could be enhanced by combining it with the conventional single-cell learning.

Another factor that should be taken into considerations is the general approaches for interpretability, which can be realized by taking advantages of a series of methods, like the integration of knowledge, algorithm design, and visualization techniques. For instance, material knowledge can be integrated with ML to enhance the model generalization. Besides, design of the algorithm can be conducted. An interpretable ML combining the RF model and the Shapley additive explanation (SHAP) analysis has been proposed to accelerate the identification of the critical factors that make influence on the formation energy among the complex variables introduced by doping in Ni-rich layered oxide cathodes [165].

The experimental validation of these predictions by quantitative metrics is a critical procedure for these AI systems. It is ideal to conduct the validation by comparing the performance of different models on multiple datasets with a series of quantitative metrics, like root-mean-squared error (r.m.s.e.), mean absolute error (MAE), and mean absolute percentage error (MAPE), for which the smaller deviation between the predicted value and the true value indicates the stronger predictive ability. Besides, it is better to make the comparison between the proposed model and the other models, and different datasets are expected to be used. Moreover, it is also of importance to evaluate the reliability in dealing with critical and complexed tasks in the real-world applications. Additionally, the error range should also be taken into considerations to make a full evaluation of these predictions. For instance, in an attempt to design the DL framework for making a prediction of battery lifetime, the performance comparisons among the proposed models and other models have been made by using a series of indexes, like r.m.s.e. and MAE with the error range being indicated, as well [147]. It was noticeable that error bars when combined with other statistical quantities such as standard deviation could visually represent the extent of variation of the corresponding models across multiple trials. In this case, when comparing the performance of different models, error bars enabled a more comprehensive assessment of the strengths and weaknesses of various models.

### Autonomous Laboratory Validation

Material synthesis is featured with complexity with many factors like the kinetics and thermodynamic stability of materials, the synthesis routes, synthetic methods, and precursor species being taken into considerations [87]. The automated synthesis and characterization are important parts of the closed-loop AI systems for materials [166]. Robotic laboratories can be served as excellent platforms for data-driven experimental synthesis science to guide human and robotic chemists [88, 167]. It is worthwhile mentioning that the autonomous workflows based on liquid handling have been successfully realized for organic chemistry [131–134].

In regard to dealing with and characterizing solid inorganic powders, an autonomous laboratory has been built for the accelerated preparation of new materials [101]. The A-Lab performed experiments with three integrated stations for different tasks, including sample preparation, heating and characterization, and robotic arms were responsible for transferring samples and labware, which is illustrated in Fig. 6i. It turned out that the A-Lab was capable of realizing 41 novel compounds from a set of 58 targets with a success rate of 71% after continuous operating over 17 days.

It was pointed out that both the high reproducibility and throughput could be realized by the robotic laboratory simultaneously [88]. For instance, a robotic laboratory was taken advantages of to carry out the large-scale validation of precursor selection. As shown in Fig. 6j, k, a full ceramic synthesis workflow could be accomplished automatedly by a robotic arm, including precursor preparation, ball milling, oven firing, and product characterization. As illustrated in Fig. 6l, exploration of synthesis hypotheses in large-scale could be achieved by robotic laboratory, while it took a lot of human experimentalists many years to finish such intense work. Furthermore, it was difficult to weight the throughput and reproducibility for large-scale human work. In contrast, both the high reproducibility and throughput could be realized simultaneously by the robotic laboratory for the reason that it was possible for a robotic laboratory to produce single-source experimental data with high reproducibility, which is shown in Fig. 6m. It turned out that a comprehensive amount of synthesis hypotheses was managed to be explored rapidly and reproducibly by the robotic laboratory which could be served as a novel platform for the data-driven synthesis science.

Progress has been made to apply autonomy in a diversity of aspects in materials research, with robotic, the optimization of material yield, the improvement of photovoltaic performance, and the enhancement of photocatalysis activity included. However, in contrast to human researchers who have rich background knowledge facilitating their decision-making, some limitations still exist for the A-Lab in these aspects, and therefore, a fusion of encoded domain knowledge, the access to various data sources, and active learning are especially important for the autonomy. In addition to this issue, discrepancies between the current predictions and the experimental outcomes are needed to be further addressed.

Another challenge that is met for the AI applied in material science is that there is gap between the predicted results and the feasibility of the experiment. Such an issue in result from a series of aspects. For example, in the early stage of new material research and development, the data available is scarce, and there exists the problem of overfitting or underfitting. Besides, the economic imbalance between the verification system and the experimental cost can also lead to the gap between the predicted results and the experiments. These gaps are usually in high relationship with the cognitive gap among data, models, and experiments. Cross-scale data fusion (combining atomic simulation with macroscopic characterization), the human-machine collaborative experimental design (reinforcement learning and domain experts), and other measures can be taken for narrowing the gap between the predictions and practice.

## Strategies to Design AI Systems for Materials with Enhanced Performance

### For Cognition of Existing Materials

#### Existing Data Leverage

**Fig. 7:**
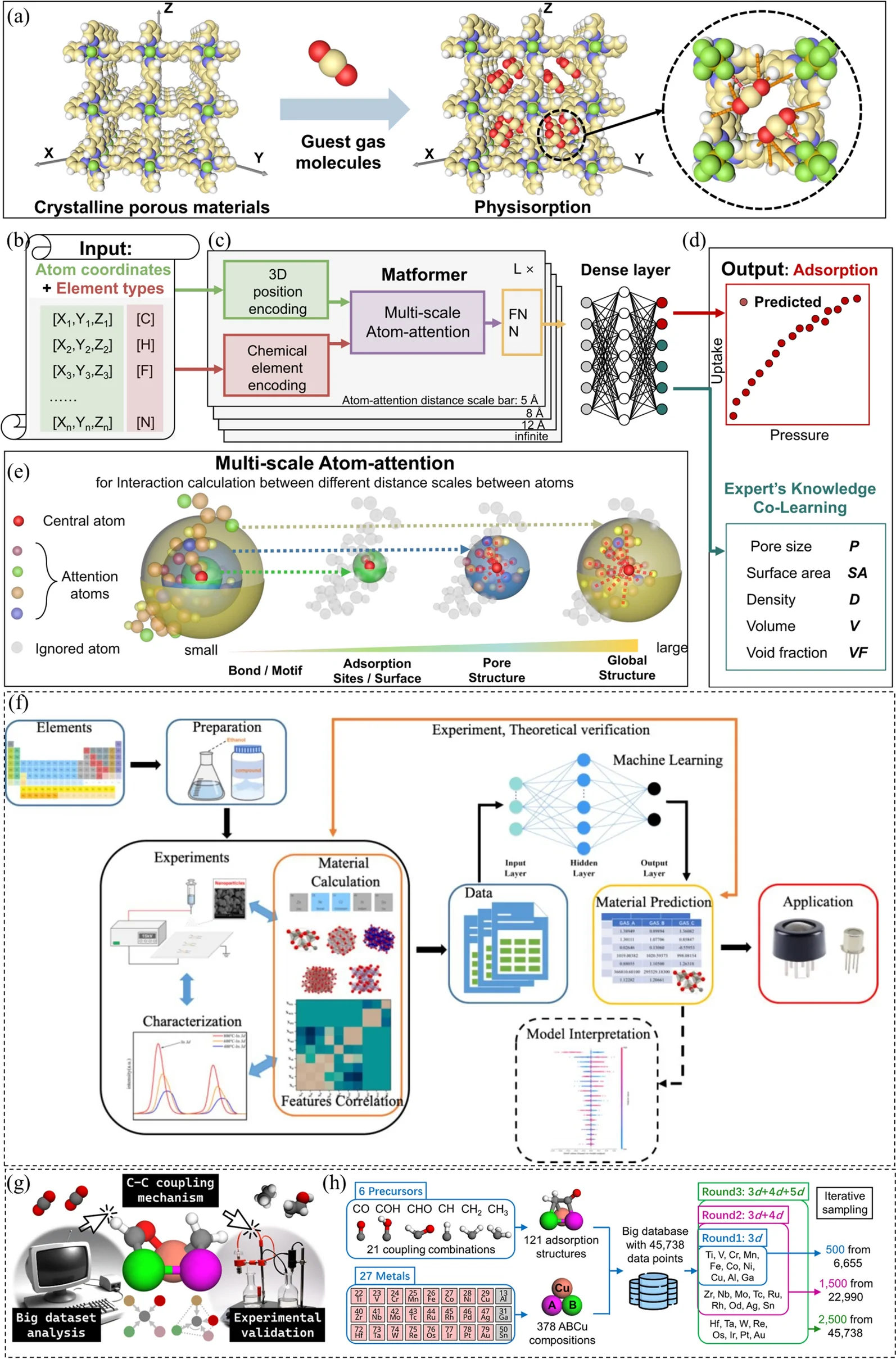
**a** Schematic of the physisorption process with the materials used for training collected from some datasets. **b** Inputs of DeepSorption. **c** Architecture of MatFormer. **d** Outputs of DeepSorption. **e** Schematic of MSA. Reproduced under the terms of the CC-BY license. [102] Copyright 2023, The Authors, published by Nature. **f** ML-enabled exploitation of gas-sensing descriptors with the computations based on experimental and characterization results. Reproduced with permission from Ref. [170]. Copyright 2024, Elsevier. **g** Schematic illustration of ML big dataset analysis, and **h** the construction and sampling of big dataset. Reproduced with permission from Ref. [171]. Copyright 2024, American Chemical Society

The materials used for training can be collected from some datasets [102]. To be specific, the knowledge of porous materials and their physisorption behaviors are beneficial for the ML-enabled rapid discovery of materials with desired adsorption properties [168, 169]. For instance, in order to develop a spatial atom interaction learning network for the prediction of gas adsorption (Fig. 7a), computation-ready, experimental MOF (CoREMOF), hypothetical MOFs (hMOF) and EXPMOF datasets were used. To be specific, CoREMOF dataset includes over 11,000 computation-ready and experimental three-dimensional metal-organic frameworks (MOFs) obtained from Cambridge Structural Dataset and Web of Science, while hMOF dataset includes over 300,000 hypothetical MOFs. Additionally, the EXPMOF dataset is from experiments. In this case, the original data of crystalline materials could be directly used as the input of DeepSorption without information loss, which is illustrated in Fig. 7b, and the outputs including gas adsorption isotherms could then be obtained (Fig. 7d). It is worthwhile mentioning that targeted data processing methods were developed. The homemade MatFormer (Fig. 7c) featured with Multi-scale Atom-attention (MSA) was used to process crystalline material data for the reason that it was managed to provide conception of the interactions between different defined atoms, which is shown in Fig. 7e. The judgment of the interatomic interaction at different scales could be promoted by the exchange of information between atom pairs in different distances.

The computations can also be based on experimental and characterization results [170]. Recently, ML has been exploited for exploitation of gas-sensing descriptors, which can predict the gas-sensing performance of oxides (Fig. 7f). To be specific, data were obtained for five pristine oxides that were commonly applied as gas sensors. The input features were based on the characterization, computational results, and physical properties of the materials and gas molecules. The importance of the features was ranked, and six important features were proposed as the descriptors. It was worthwhile mentioning that many properties such as morphology, band structure, and surface composition could exert significant effects on gas-sensing reaction, and therefore, the oxides were characterized by a series of tests.

For some complex cases, it is necessary to construct big dataset to fully reveal the underlying mechanisms and the optimal direction for material design [171]. For example, carbon–carbon (C–C) coupling is of importance in the electrocatalytic reduction of CO_2_ in order to produce green chemical. However, the reaction network is usually complex. To address this problem, big data analysis was introduced into the computational screening of electrocatalysts for complex C–C coupling reaction networks (Fig. 7g). It was worthwhile mentioning that a big dataset with over 45,000 data points was constructed, covering all possible coupling combinations of six precursor species as well as adsorption configurations on the active site. As illustrated in Fig. 7h, 378 adsorption substrates made use of ABCu triatom active sites with 27 metal replacements for A and B, and iterative sampling was taken advantages of to obtain the training set for ML.

In addition to the construction of big dataset, some methods have been proposed for the cases in which the dataset is quantitatively limited and qualitatively biased [108]. For instance, a ML framework was developed for the highly generalizable prediction of temperature-dependent Flory–Huggins χ parameters. The experimentally observed χ parameters for 1190 samples were used for training the model. However, this dataset was lack of chemical diversity, and the experimental χ parameters were biased, which limited the application of the model. Another significant bias existed in the aspect that some observable χ parameters would be given only for polymer-solvent molecules in a miscible state due to technical limitations. It could be observed that the majority in the experimental χ parameter dataset was consisted of soluble samples. Specifically, it was difficult to realize experimentally determining χ parameters for an immiscible polymer-solvent system in which no single phase appeared. In order to address this issue, two auxiliary datasets were constructed, among which one was extracted from PoLyInfo with a list of 29,777 soluble and insoluble polymer–solvent pairs and the other was an in-house dataset obtained by making use of quantum chemistry calculations with COSMO-RS. It was proposed that polymers and solvents in PoLyInfo were distributed over a wider chemical space. It was verified that the applicability domain of the model was managed to be successfully expanded by learning with the two additional large datasets.

#### Structure and Property Prediction

**Fig. 8:**
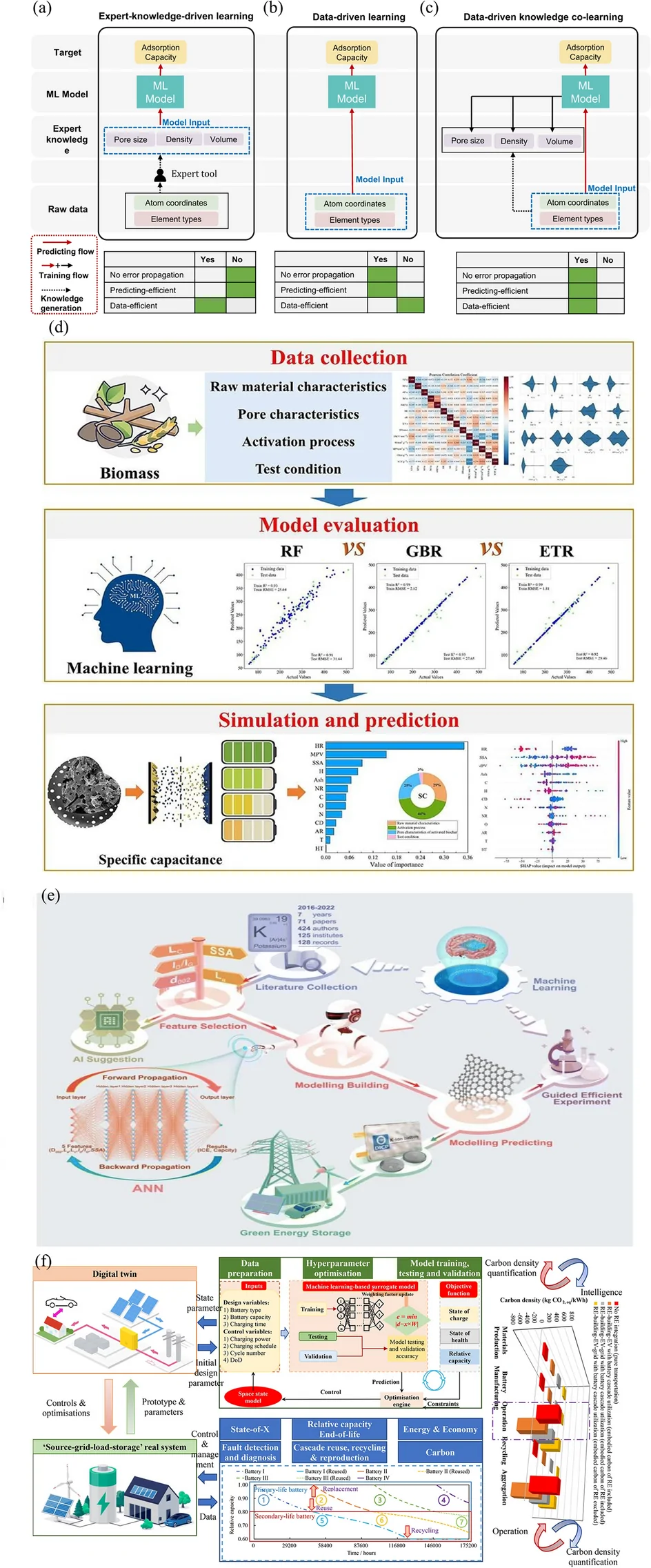
Schematic illustration of **a** Expert-knowledge-driven learning, **b** Data-driven learning, and **c** Data-driven knowledge co-learning. Reproduced under the terms of the CC-BY license. [102] Copyright 2023, The Authors, published by Nature. **d** Schematic illustration of ML enabled the optimal preparation method of biochar-based electrodes. Reproduced with permission from Ref. [172]. Copyright 2024, Elsevier. **e** Schematic relationship between different parameters and performances. Reproduced with permission from Ref. [114]. Copyright 2023, The Royal Society of Chemistry. **f** Schematic illustration of frontier digital twin-based battery sustainability platform. Reproduced with permission from Ref. [173]. Copyright 2024, Elsevier

As to ML for structure or property prediction of the existing materials, it is also essential to make selections of different ML methods. One case in point was that knowledge co-learning (KCL) was chosen when developing a spatial atom interaction learning network [102]. It was proved that the KCL could enhance the convergence of the model in the structure-adsorption space establishment with the assistance from the expert knowledge in the auxiliary tasks by the comparison of the Expert-knowledge-driven learning (Fig. 8a), Data-driven learning (Fig. 8b), and Data-driven knowledge co-learning (Fig. 8c), and therefore, the prediction accuracy could be improved.

Another case in point was that three ML models were developed for the optimal preparation of biochar-based electrodes [172]. As illustrated in Fig. 8d, 14 key parameters from recent articles focused on the preparation of activated biochar-based supercapacitor electrode with urea as the nitrogen source and KOH as the activator were collected. Three classic ML prediction models, with RF, GBR, and extra tree regression (ETR) included, were made used of to make an exploration of the response relationship between various factors and the energy storage properties. It turned out that the GBR demonstrated the best prediction performance with an *R*^2^ value of 0.93.

Methods have been come up with to handle the issue of limited data supplying in the primary tasks [108]. For instance, in a neural network architecture developed for the prediction of the χ parameter with limited data providing for the parameters in the primary task, multitask learning was applied, in which different related tasks with common underlying mechanisms shared were learned simultaneously via a unified model. It was clarified that the multitask learning was able to boost the predictive performance by leveraging and transferring feature representations learned from two auxiliary tasks.

ML can be used to predict the relationship between different parameters and performance with the suitable models [114]. For instance, a strategy to construct hierarchical porous sponge-like carbon was launched for advanced potassium-ion batteries, in which cases ML was taken advantages of to offer further evidence of the excellent performance. Papers focused on layered carbon materials for potassium batteries were made use of to construct the structural parameter performance database. The complete initial coulombic efficiency (ICE) and capacity structural parameters performance database were input into ANN, which is demonstrated in Fig. 8e. It was verified that the predicted capacity and ICE were almost equal to the experimental values.

#### Experimental Validation

The prediction capacities for structures or properties are usually examined by experiments comprehensively. It is noticeable that the prediction performance could then be evaluated from various aspects and in a diversity of conditions [102]. For example, the spatial atom interaction learning network was employed for prediction of gas adsorption, and it was verified that the predicted gas uptake was consistent with the actual value on CoREMOF-CO_2_ and hMOF-CO_2_. In contrast to the other models, there absolute errors were much smaller and more distributed centralized for DeepSorption. Furthermore, higher coefficient of determination (*R*^2^) values could be realized. It turned out that both the highest *R*^2^ value and the lowest MAE could be achieved by DeepSorption compared with the other models.

As a powerful tool for material research, AI has been integrated with other advanced technologies for the formation of more complex platforms [173]. For instance, a cross-scale multi-stage analytic platform featured with inter-disciplinary and trans-disciplinary was developed for the lifecycle carbon intensity investigation of electrochemical batteries, including battery materials, charging/discharging behaviors, recycling, and reproduction (Fig. 8f). ML was applied to address the issues that the collected data from controlled test conditions in the laboratory were not managed to represent various real application scenarios, and the state-of-charge prediction could be made. Besides, ML-assisted computation could promote the sustainability and climate adaption for this framework. Furthermore, by taking advantages of the digital twin, the performance estimation could be cost-saving and time-efficient.

In addition to the theoretical approaches, how these AI systems for material discovery and synthesis make contribution to the real-world examples with experimental implementation and practical validation is another valuable aspect to be explored, and more researches have been carried out focused on how to utilize these systems to address the practical issues. The lithium-ion batteries, which are featured with high energy densities and low production costs, have drawn great attention in many modern industries, serving as renewable energy solutions for many fields, like electric vehicles.

It is worthwhile mentioning that the combination of AI with battery lifetime prediction is also one of the research hotspots, since the capacity of these batteries fades inevitably with cyclic operations, which is attributed to the intrinsic electrochemical mechanisms. Great challenges have been met due to a variety of factors that influence the complex battery capacity degradation, like electrode materials, cycling protocols, ambient temperatures, and so on. Some cutting-edge researches have been conducted with the effective solutions to address these issues. For instance, a DL framework, BatLiNet, which was designed to predict battery lifetime reliably across a variety of aging conditions, was proposed [147]. In contrast to the traditional models which solely focused on individual cells, this framework adopted inter-cell learning which contrasted pairs of battery cells for discerning lifetime differences. It was noticeable that the experimental results, derived from a broad spectrum of aging conditions, verified its superior accuracy and robustness in this research when comparing to other existing models. In addition to the design of the frameworks, efforts have also been made to meet the challenges proposed by the intersection of electrochemical science and ML, and accordingly, an open-source platform with data preprocessing, feature extraction, and the implementation of both conventional and state-of-the art models integrated has been developed, which aims to provide a collaborative platform on which experts from diverse specializations can contribute their own efforts [174].

### For Discovery of New Materials

#### Excavating the Existing Data

**Fig. 9:**
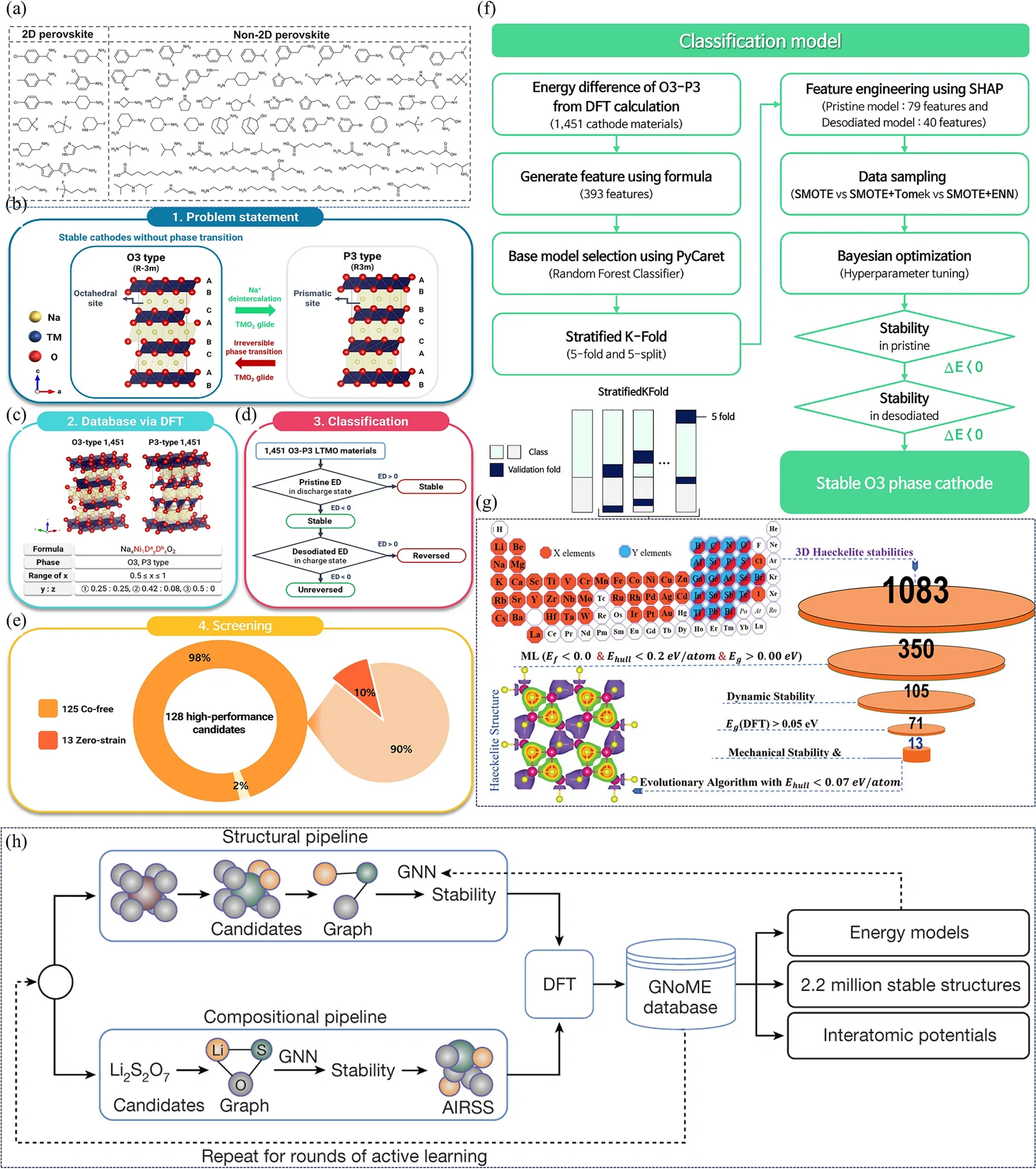
**a** Synthesis results of high-throughput experiments. Reproduced under the terms of the CC-BY license. [87] Copyright 2024, The Authors, published by Nature. Schematic illustration of **b** problem statement, **c** database construction, **d** ML classification, and **e** screening result. **f** Schematic of the classification model. Reproduced with permission from Ref. [18]. Copyright 2024, Elsevier. **g** Workflow of ML for new Haeckelite compounds discovery. Reproduced with permission from Ref. [52]. Copyright 2024, Wiley. **h** Schematic illustration of two frameworks to for generation and filtration. Reproduced under the terms of the CC-BY license. [51] Copyright 2023, The Authors, published by Nature

The dataset used for training is the cornerstone of ML models [175]. The experimental synthesis data provided by studies serve as important resources for the material synthesis. However, only successful cases are usually included in these studies, resulting in the imbalanced distribution of data category. Another important resource is from the first-principles calculations. Besides, previous studies and extensive laboratory experience can offer valuable intuitions for the preparation of new materials. For instance, in an attempt to explore the synthesis feasibility of two-dimensional silver/bismuth (2D AgBi) iodide perovskites, organic spacers from both the previously reported 2D perovskites and the chemical intuitions were exploited [87]. The high-throughput experiments (HTE) were made use of to acquire the material dataset. It was proved that only 13 kinds of organic spacers were able to form 2D AgBi iodide perovskite structures, and the organic spacers were sorted into ‘2D perovskite’ and ‘non-2D perovskite’ accordingly, which is shown in Fig. 9a.

DFT calculations which are quantum mechanical theory-based tools also play an important role in high-throughput computational material design for the reason that they can characterize material properties and produce data directly [18]. For example, in an attempt to develop Co-free and low strain cathode materials for sodium-ion batteries with the assistance of ML (Fig. 9b), 1451 O3 and P3 layered transition metal oxides (LTMOs) were generated via DFT calculations, which is illustrated in Fig. 9c. The classification ML models were then constructed to evaluate the structural stability and phase transition (Fig. 9d), leading to the identification of 128 highly reversible high-performance cathode material candidates (Fig. 9e). In this study, endeavors have been made to solve the problem of imbalanced data by a data sampling technique. In particular, a stratified k-fold importing data hierarchically from every class were taken advantages for the construction of a balanced train set, which is shown in Fig. 9f. Given the fact that there were not enough data, it was conducted in fivefold (train set/validation set = 8:2), so that the number of validation set could be guaranteed.

Although there are both positive and negative material data in the datasets from HTE, subjective preferences still exist. As a result, it is difficult for ML to obtain reliable conclusions. Efforts have been made to address this issue. For example, in the framework to guide the experimental synthesis of two-dimensional perovskites, data-mining approaches were taken advantages of to identify the applicable subdomains for ML models, and then, models were trained on the identified subdomain, which showed more distinctive descriptors than models training on the whole biased dataset [87]. To be specific, subgroup discovery was used to get the suitable subdomains for ML models. It turned out that the molecular weight and the third ordered kappa index were the two descriptors standing out since they had a high correlation with the synthesis feasibility. Besides, based on the derivation of the rigid sphere model, the width of organic spacers was also of importance for the structural stability. In addition, 2D projections of this 3D data distribution map were generated. The distribution of 2D perovskites and non-2D perovskites was balanced in the determined specific subdomain.

A series of steps are taken for the preparation and preprocessing of data. In an attempt to discover new Haeckelite compounds for optoelectronic devices with the assistant from ML, data preparation and preprocessing were carried out [52], and the workflow of screening and predicting is shown in Fig. 9g. To be specific, the selected chemical space of the ML dataset with X and Y elements was demonstrated in red and blue, and 1083 square-octagon XY form structures were created. After that, the compounds with altered symmetry and duplication were removed. A base dataset for the train structure was selected, which was both quantitatively and qualitatively accurate when compared with other benchmarks, and then, more compatible optimal features to predict each target with high accuracy and minimum error were constructed. It turned out that 350 materials were got after the investigation of the formation energy, bandgap, and convex hull energy by comparing them with the experimental targets using ML, and 13 semiconducting Haeckelite structures were obtained after the calculations of electronic structures, dynamic stability, and the multistep evolutionary.

It is noticeable that in some cases the space of possible materials is far too large, and it is difficult to sample in an unbiased manner [51]. Under the condition that there is no reliable model available to approximate the energy of candidates cheaply, the substitution of similar ions or enumeration of prototypes has been made use of according to chemical intuition. There still exists limitations in regard to the diversity of candidates even though the search efficiency has been improved, and therefore, it is critical to build new method to make more diverse candidates available. In an attempt to solve this problem, two frameworks were taken advantages of to generate and filtrate these candidates, which is illustrated in Fig. 9h. Particularly, the structural candidates were managed to be generated by modifying available crystals. It was worthwhile mentioning that efforts have been made to augment the set of substitutions by means of adjusting ionic substitution probabilities to give priority to discovery. Moreover, newly proposed symmetry aware partial substitutions (SAPS) were used to enable incomplete replacement efficiently. As to the second framework, compositional models could predict stability free of structural information. The graph networks for materials exploration (GNoME) were trained on available data to filter candidate structures. It was worthwhile mentioning that for the models, the crystal definition, which encoded the lattice, structure, and atom definitions, was served as the input. In particular, each atom was represented as a single node in the graph, and edges were defined on the occasion where the interatomic distance was less than the defined threshold. Nodes were embedded by atom type and edge, and they were embedded on the basis of the interatomic distance. A global feature that was connected in the graph representation to all nodes was also made use of. At every step of the GNN, neighboring nodes and edge features were aggregated, and they were utilized to update the corresponding representations of nodes, edges, or globals individually. After 3-6 layers of message passing, an output layer projected the global vector so as to obtain an estimate of the energy. It was verified that almost an order of magnitude larger than previous work could be achieved via GNoME.

#### Screening for Excellent Performance and High Synthesis Feasibility

**Fig. 10:**
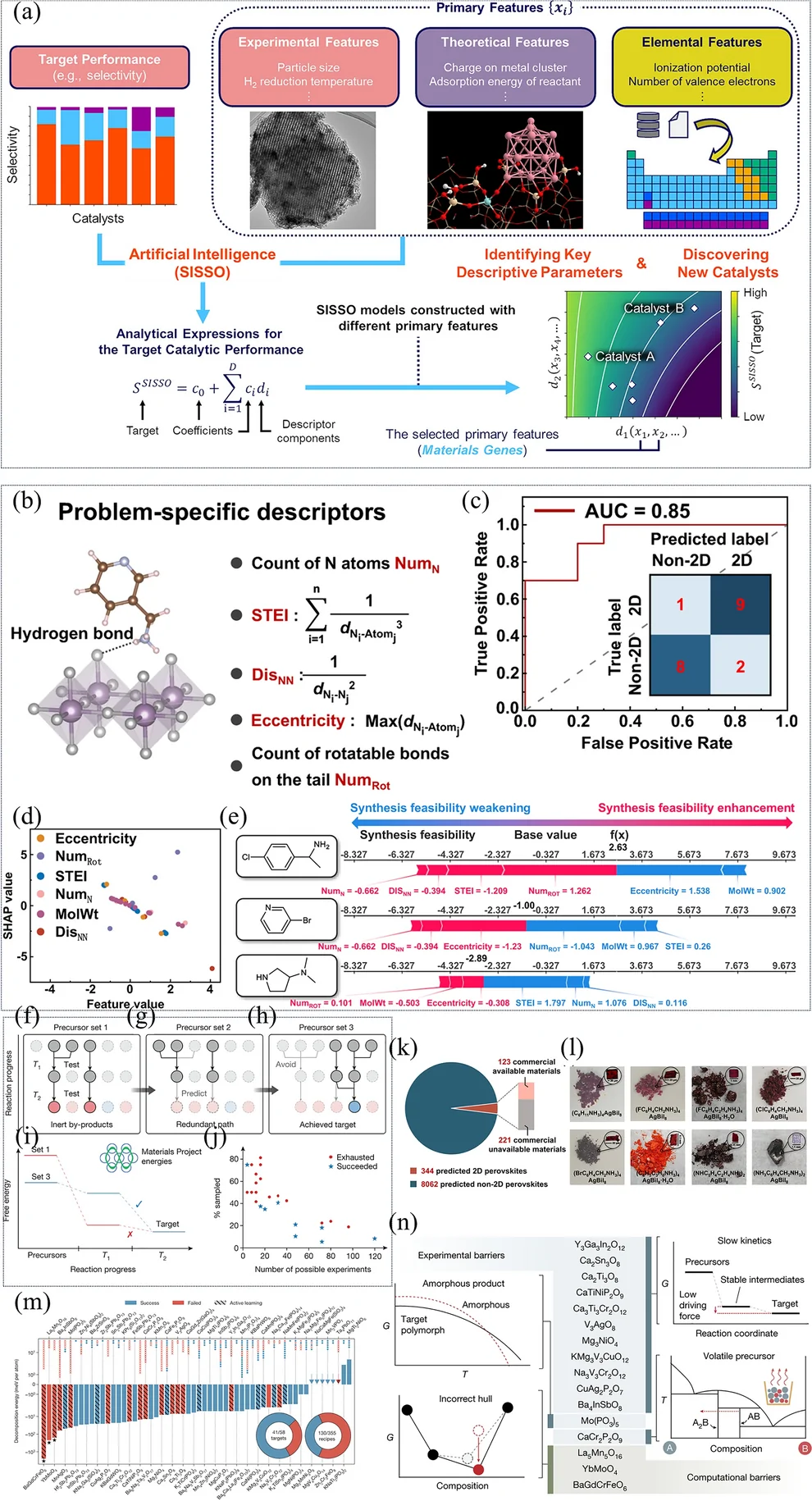
**a** Schematic illustration of SISSO AI for integration of materials parameters. Reproduced under the terms of the CC-BY license. [91] Copyright 2024, The Authors, published by American Chemical Society. **b** Schematic of the problem specific descriptors. **c** Receiver operating characteristic (ROC) curve and confusion matrix. **d** SHAP values for the six features. **e** Predicted synthesis feasibility. Reproduced under the terms of the CC-BY license. [87] Copyright 2024, The Authors, published by Nature. **f** Schematic of how to estimate which pairwise reactions occurred from a failed attempt. **g** Schematic of new precursors recommended by substituting at precursor relevant to the unfavorable pairwise reaction and **h** schematic illustration of the successful precursor set. **i** Free energy at each step. **j** Number of experiments for exhausting all unique reaction paths of each target or identifying an optimal path. Reproduced under the terms of the CC-BY license. [101] Copyright 2023, The Authors, published by Nature. **k** Schematic of screening for the prediction set, and **l** the experiment validation. Reproduced under the terms of the CC-BY license. [87] Copyright 2024, The Authors, published by Nature. **m** Experimental outcome. **n** Barriers for the synthesis of the targets. Reproduced under the terms of the CC-BY license. [101] Copyright 2023, The Authors, published by Nature

AI can meet the demand for screening the materials with excellent properties and high synthesis feasibilities, which has significantly promoted the discovery and realization of new materials with excellent performance for various applications, such as catalysts, lithium-ion batteries, and perovskite solar cells [91]. Endeavors have been made to identify the key parameters of materials and suggest the new candidates which can be verified experimentally. For instance, in an attempt to address the issue about the catalyst design existing in the aspect that their performance was influenced by an intricate interplay of various multiscale factors, like the chemical reactions on surfaces, the type of support materials, and the material restructuring during the catalytic period, symbolic-regression AI was taken advantages of to extract the key physicochemical parameters related to the performance successfully (Fig. 10a). It was proposed that the Sure-Independence Screening and Sparsifying Operator (SISSO) had been introduced into data-centric methods for heterogeneous catalysis with a series of advantages [176, 177]. To be specific, analytical expressions related to the target catalytic performance could be identified by SISSO with few key parameters out of many offered parameters, which were regarded as materials genes to demonstrate the catalytic function of the materials. The intricate correlations between small datasets could be demonstrated with an immense amount of candidate functions being under consideration during the analysis process. In particular, the theoretical, experimental, and elemental parameters as the primary features were made use of to efficiently model the catalytic performance. The analytical expressions were pointed out. It was noticeable that the key descriptive parameters deemed as materials genes which were in close relationship with the property were obtained. By using the AI model with low costs, new additives could also be suggested, which promoted the discovery of new catalysts with high performance.

When it comes to new material synthesis, a diversity of factors should be taken into consideration, such as the precursors, the by-product, the feasibility of experimental conditions, and the availability of experimental raw materials, which makes it a more complex and time-consuming task for chemists due to the limitation of experimental instruments and the heavy workload. As a result, only a small subset of the potential conditions can be evaluated with a rather small proportion of theoretically predicted materials being synthesized successfully, and this work heavily relies on the experience of chemists. In this case, the data-driven techniques can be applied for screening out materials with high synthesis feasibilities to provide guidance for the material synthesis [87]. It is worthwhile mentioning that efforts have also been made to extract meaningful physical and chemical insights from trained ML models in order to have a better understand the ML predictions. For instance, ML techniques were made use of to screen two-dimensional hybrid organic–inorganic perovskites (2D HOIPs) with high synthesis feasibility rapidly. A lot of distinctive descriptors relevant to the synthesis feasibility were developed (Fig. 10b). The support vector classification (SVC) algorithm was applied to develop the equation for the synthesis feasibility. The receiver operating characteristic (ROC) curve and confusion matrix which were used to evaluate the accuracy and the error are shown in Fig. 10c, and the area under the ROC curve was as high as 85% where only 1 out of 10 molecules was misclassified. The marginal contribution of individual descriptors was analyzed by SHAP analysis, the result of which demonstrated that the number of rotational bonds in the alkyl tail (Num_Rot_) was the most important factor for the synthesis feasibility. The relationships between the feature values and SHAP values are illustrated in Fig. 10d. A positive SHAP value indicated that the feature led to high synthesis feasibility, while a negative one resulted in low synthesis feasibility. The predicted synthesis feasibility is demonstrated in Fig. 10e.

ML also plays an important role in planning and interpreting the outcomes of experiments, which is critical to bridge the gap between computational screening and experimental realization of new materials. For instance, by combining with other tools, including computations and historical data getting from the studies, it was possible for ML model to provide up to five initial synthesis recipes for the proposed compounds [101]. Specifically, target ‘similarity’ was evaluated by means of natural-language processing of a large database extracted from the literature, during which process the behaviors of a human carrying out the initial synthesis referred to known related materials were mimicked [129]. Furthermore, the active learning was made use of to identify synthesis routes with improved yield. A database of pairwise reactions was continuously constructed by the autonomous laboratory from its experiments, which made it possible for the products of some recipes to be inferred (Fig. 10f, g). As a result, the search space of possible synthesis recipes could be reduced by up to 80% as many precursor sets reacted to form the same intermediates (Fig. 10j). Besides, knowledge of reaction pathways was used to give priority to the intermediates with large driving force to form the target which could be obtained from the formation energies provided by the Materials Project (Fig. 10h, i).

#### Experimental Realization

Experiments are usually carried out in order to realize the new materials either by researchers or by autonomous laboratories. Some equations obtained can be used to predict unexplored molecules. For instance, 344 2D perovskites with high synthesis feasibility were screened out [87], which is illustrated in Fig. 10k. Given the fact that organic spacers in the prediction set were gathered from PubChem, some amines were commercial unavailable, leading to only 123 predicted 2D AgBi iodide perovskites to be possible for further experimental synthesis. Experiment validation was then carried out. The synthetic chemical reagents were used as received. In order to eliminate the competing Bi-based phases, an excess amount of Ag_2_CO_3_ was made use of. For instance, an amount of Ag_2_CO_3_ and Bi_2_O_3_ were dissolved in concentrated hydroiodic acid with the heating temperature of 393 K. 1-(4-chlorophenyl) ethan-1- amine was added to H_3_PO_2_ in another beaker, and then, the two solutions were mixed, which was allowed to evaporate at the hot plate with the temperature of 323 K for a day. Finally, brownish red crystals precipitated at the bottom of the beaker could be obtained successfully. As for validation of the ML model, 13 commercially available organic spacers without hydroxyl and ether were unbiased selected, and 8 of 13 predicted 2D AgBi iodide perovskites which showed high synthesis feasibility were successfully synthesized with the success rate of 61.5%, indicating a much higher success rate than chemical intuition (16.4%) (Fig. 10l). It was worthwhile mentioning that the repeatability and stability are critical to the experiment validation, and some measures can be taken to ensure the repeatability. For instance, in this case, ten individual repetitions of the synthesis process for (NH_2_C_5_H_8_F_2_)_4_AgBiI_8_ were implemented to make an assess of the experimental reproducibility of the synthesis experiments.

An autonomous laboratory was designed for the accelerated synthesis of novel materials integrated with computations, historical data, ML, and robots to conduct experiments [101]. Recipes for synthesis of the novel materials were tested using a robotic laboratory, which was managed to perform the powder dosing, sample heating, and product characterization. The experimental outcome is demonstrated in Fig. 10m. It is proposed that the robotic experimentation efficiently accelerated the experimental synthesis of materials. The high success rate also verified that it was possible for comprehensive ab initio calculations to discover novel and synthesizable materials effectively.

Some further analysis can be carried out about the barriers for the synthesis of the targets [101]. For example, for the 17 of the 58 targets evaluated by the A-Lab which were not realized even though active learning was taken advantages of, the ‘failure modes’ were classified as experimental barriers which were marked as blue and computational barriers which were marked as green in Fig. 10n. The continued efforts have been made by researchers to create new materials experimentally in order to offer a way to validate the AI findings.

## Design Consideration of the AI Systems for New Materials

### To be More Autonomous

The intelligent systems are expected to be more autonomous with the capability to interpret data and make decisions. Autonomy has realized from some aspects in regard to materials science. It is widely considered that a fusion of encoded domain knowledge, access to a variety of data sources, and active learning are critical for the accomplishment of enhanced autonomy [178, 179].

**Fig. 11:**
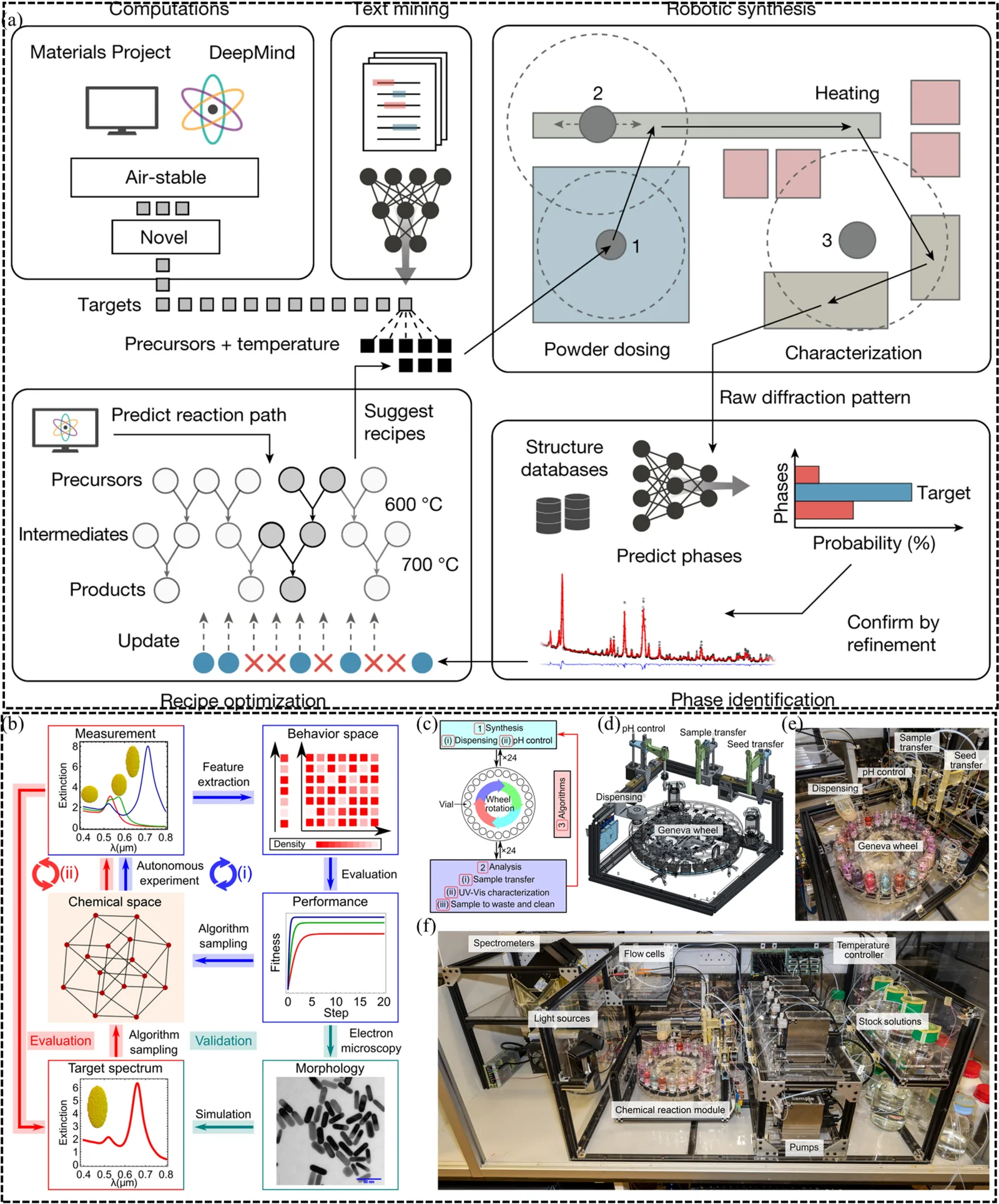
**a** Schematic of A-Lab to discover materials autonomously. Reproduced under the terms of the CC-BY license. [101] Copyright 2023, The Authors, published by Nature. **b** Closed-loop approach of exploration and optimization. **c** Workflow of the closed loop. **d and e** CAD design and the experimental setup. **f** Overall setup of the platform. Reproduced under the terms of the CC-BY license. [89] Copyright 2022, The Authors, published by American Association for the Advancement of Science

One case in point was that an autonomous laboratory was successfully constructed which was managed to realize 41 novel compounds after more than 17 days of continuous operation [101] (Fig. 11a). To be specific, the targets which were air-stable and unreported were identified via DFT-calculated convex hulls consisting of ground states from the Materials Project and Google DeepMind, after which the synthesis recipes were pointed out by means of ML models that were trained on synthesis data from the studies. The recipes were then tested via a robotic laboratory through the powder dosing, sample heating, and characterization procedures. Phase purity was evaluated via X-ray diffraction (XRD), which was then analyzed by ML models trained on structures from the Materials Project and the Inorganic Crystal Structure Database (ICSD). In particular, both the phase and weight fractions of the synthesis products were extracted from their XRD patterns by probabilistic ML models that were trained on experimental structures from the ICSD. By inverting the container, the powder was dispensed through the mesh onto an XRD sample holder, after which it was flattened with an acrylic disk. The flattened sample was transferred into the diffractometer for X-ray measurements with 8-min scans that range from 2*θ* = 10° to 100°. For n given XRD pattern got from an unknown sample, XRD-Auto Analyzer was utilized to recognize the constituent phases, and their weight fractions were also estimated. In the cases where high (> 50%) target yield was not achieved, new synthesis recipes would then be proposed by means of an active learning algorithm. It was noticeable that the whole sequence was fully automated.

Both the exploration and optimization were able to be realized via a chemical robot for the autonomous synthesis of nanomaterials [89] (Fig. 11b). Particularly, as for the exploration mode, the structural diversity was accomplished via searching for diversity in the behavior space. The fitness was evaluated based on peak prominence and broadness correlated with the yield and mono-dispersity. A new batch of experiments was generated from previous synthetic conditions for the realization of higher-performance samples. As to the optimization cycle, the target spectrum was defined via the extinction spectrum simulation of the nanoparticle with the shape derived from electron micrographs. The similarity to the target spectrum and the sampling density in the synthetic space were taken into consideration by the algorithm in order to come up with multiple optimal conditions. The workflow of the closed loop with the procedures of synthesis, analysis, and design of new experiments is illustrated in Fig. 11c. A chemical reaction module that was able to perform parallel synthesis with up to 24 reactors was served as the core robotic hardware. The rotation of the Geneva wheel was taken advantages of to carry out the synthesis efficiently. The liquid handling, pH control, sample transfer, and spectroscopic analysis could be successfully conducted. CAD design and the experimental setup are shown in Fig. 11d, e. The detailed setup of the autonomous platform for the exploration and optimization of the nanomaterials is demonstrated in Fig. 11f.

### To be More Universally Applicable

**Fig. 12:**
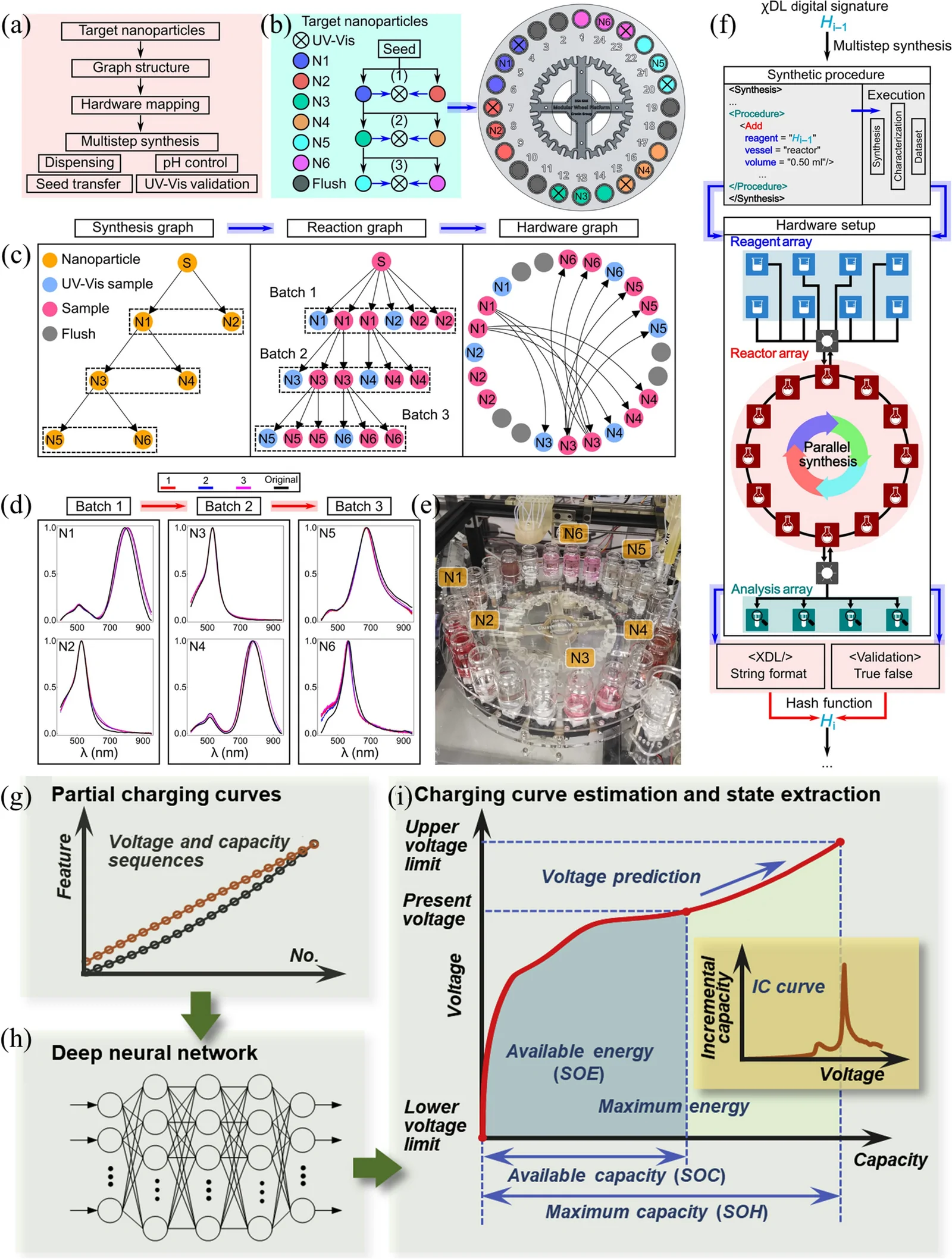
**a** Workflow of the autonomous multistep synthesis platform. **b** Six target nanoparticles. **c** Synthesis, reaction, and hardware graph. **d** UV–Vis spectra of samples. **e** Photographs of the distributed samples. **f** Illustrations of how to generate the unique digital signatures. Reproduced under the terms of the CC-BY license. [89] Copyright 2022, The Authors, published by American Association for the Advancement of Science. **g and h** Voltage and capacity sequences gathered as the input of DNN. **i** Output of the complete charging curve. Reproduced with permission from Ref. [180]. Copyright 2021, Elsevier

For the integration of material science and data-driven techniques, it is always in high demand to provide some practical routes for typical laboratory environment even though limited experimental resources are available [87]. Additionally, for the materials highly dependent on the synthetic conditions, it is expected for the AI systems to be standard and robust, so that the high reproducibility can be realized [89]. When it comes to the guarantee of the synthesis reproducibility, certain design of the platforms should be taken into consideration, and it is also essential to conduct the characterization at each step. For example, in an attempt to construct the autonomous platform for the synthesis of high yield and monodispersed nanomaterials which were very sensitivity to the synthetic conditions with the reagent concentrations, temperature, the order of reagent addition, and many other analogous factors included, workflow of the autonomous multistep synthesis was designed [89] as in Fig. 12a. Three graphs including synthesis, reaction, and hardware were required in this case, which was demonstrated by manes of synthesizing six uniquely shaped gold nanoparticles obtained from the previous exploration (Fig. 12b). It was noted that the synthesis graph represented the multistep synthetic procedure in which each node represented a unique nanoparticle and each directed edge showed the hierarchical relation between these nanoparticles (Fig. 12c). It was worthwhile mentioning that in order to verify the reproducibility, the parallel synthesis of six gold nanoparticles was repeated three times, and the standard deviation in the UV-Vis spectra could then be obtained, which is illustrated in Fig. 12d, e. Moreover, it was proposed that the unique signatures for nanomaterials in accordance with their distinctive synthetic protocols were in high demand, and therefore, the universal chemical description language χDL was taken advantages of to create the unique digital signatures, which is demonstrated in Fig. 12f.

In order to enhance the feasibility, it is ideal for the model trained on one dataset is managed to be used for other occasions operated under different scenarios while using few training data. One case in point was that the battery charging curve prediction was able to be made by deep neural network (DNN) with 30 points collected in 10 min [180]. Particularly, a piece of the charging curve was applied as the input of a DNN. Key states could then be derived from the estimated entire charging curve. It turned out that a brand new DNN was expensive in regard to cost and time for the reason that extensive battery degradation tests for collecting new training data were required, and data in the real-world were usually incomplete and sparse. DNNs were effective to accomplish this task, which was attributing to its transfer learning feature. The transfer learning was able to resort to the similar knowledge learned from the source dataset so as to improve its performance on the target dataset, reducing the required data amount and saving computational resources. Voltage and capacity sequences gathered from any part of the charging curve could be made use of as the input to the DNN, which facilitated the collection of the input data for the real-world battery management (Fig. 12g, h). The entire constant-current charging curves could be estimated (Fig. 12i). Moreover, the proposed method was able to be quickly adapt to different batteries without much training effort.

In addition to the methods mentioned above, some other approaches can also be taken advantages of to make the intelligent systems more universally applicable. Particularly, cross-scale data fusion is useful for mapping between microstructure and macroscopic performance by combining atomic simulation with macroscopic characterization, which makes sense for the autonomy and universalization of AI systems for materials. Besides, more comprehensive factors affecting the systems are required to be taken into considerations. For example, the aggregation of most publicly available datasets was utilized for gathering various aging factors when designing the models for making battery lifetime prediction. Moreover, a diversity of dataset is taken advantages of to make an investigation focused on the adaptation of the current models to different cycling protocols.

## Perspectives

Overall, the recent development, including but not limited to AI-assisted cognizance of existing materials and AI empowered new materials discovery, is reviewed in depth. Great progress has been made in the field of AI for material science, owing to the enhanced intelligence and high-efficiency (Table 1). The elaborate design lies in every aspect ranging from data exploits to the selection of algorithm (Tables 2 and 3). Progress has been made on the data cleaning, transformation, and processing, as well as proposing the approaches to address the problems when limited data are available. The elaborate selections of models have been made to enable the accuracy, simplicity, and computation efficiency. The diverse experiments have been conducted either by chemists or by robots so that the timely and accurate validation can be guaranteed. AI has boosted the development of various functional materials applied in a diversity of field, including solar cells, nanogenerators, crystals, semiconductors, and so on. There is a growing trend toward the AI systems that are fully autonomous and universally applicable, which lead to the large-scale exploration and more abundant materials to be discovered. This review offers a keen insight into the design ideas for the AI empowered deep cognition of existing materials and fast discovery of novel materials, and some perspectives of the AI systems for materials applied in the future are proposed as follows:

1. It is ideal for input data more flexible and easier available in the real-world applications. For instance, the intelligent systems for material discovery are expected to be effective even in a standard and simple laboratory. A lot of experimental synthesis data are required by the conventional ML, which proposes big challenge for simple laboratories. Recently, the scheme has been provided for the feasibility of material synthesis in the cases where the available experimental resources are limited, which combined ML techniques with small-scale experiments to accelerate the synthesis of two-dimensional perovskites in a typical laboratory. Additionally, it is also difficult for the investigation of catalysts in which occasion the collection of a large amount of consistent experimental data is always time-consuming. This problem has been successfully solved by the SISSO that can identify potentially nonlinear and intricate correlations between small datasets with a large amount of candidate analytic functions taken into consideration. Besides, the similar issues also exist for the AI-assisted estimation of the maximum battery capacity where a complete charging/discharging curve is needed. However, the complete charge curves are hard to be obtained, since the charging process can start at various states, leading to the record of only pieces of charging/discharging curves. Accordingly, battery charging curve prediction can be made via DNN with voltage and capacity sequences collected from any part of the charging curve as the input. In the future research, more approaches are expected to be proposed to make the input data much easier to be collected in the real-world applications.
2. The accurate and comprehensive estimations and predictions about materials assisted by AI are always in high demand. (a) It is expected that the accurate prediction even for some complex issues which are strongly influenced by a series of factors can be realized. Recently, some efforts have been made for the accurate prediction of gas adsorption by the DeepSorption which is a data-driven network with a KCL module, even though every piece of subtle structural information is important for the correct description of adsorption properties. (b) Further to the accurate prediction, the comprehensive reflection of materials is also essential. For the degradation monitoring of battery which calls for the evaluation of battery states over the battery life, method has been developed so that the multiple states can be comprehensively reflected by means of using signals collected from daily battery operation. In the future work, more efforts can be made focused on the fully estimation of the materials with the data obtained from their daily operations when it is required by the practical application.
3. Endeavors can be made on enhancing the transparency in the predictions of the ML models, which can facilitate the extracting of physical and chemical insights. (a) It is critical to select the models with balanced predictive accuracy and interpretability, which can promote the development of new theories, for the reason that knowledge obtained from the interpretable ML models can accelerate the scientific understanding. For instance, more reliable explanations can be offered by inherently interpretable ML models with functions that can be approximated well via simpler functions concerned with priori knowledge, playing a more important role in extrapolating. Universal ML has been developed for the synthesis of two-dimensional perovskites recently, which can underlie the structure–property relationship in the HTE. (b) Moreover, the unveiling of predictive insights for properties by ML can further facilitate the optimization of the devices with enhanced performance. Efforts have also been made to unveil the complex relationships between piezoelectricity and TENG performance via the principal component analysis (PCA). The in-depth understanding of output offers insights into the energy conversion efficiency, and then the nanogenerator performance can be optimized.
4. The explorations on more abundant types and properties of functional materials assisted by AI are expected to be made, so as to make full use of AI. a) More factors and indexes are expected to be investigated by the data-driven techniques in material science. The synthesizability of novel materials with a variety of oxides and phosphates included has been explored by an autonomous laboratory combining computations, historical data, and ML. In the future, more factors with microstructures of materials and performances of various devices can be taken into consideration. b) In addition to inorganic materials and metals, AI can also be applied in the research of organic materials and composites. Multitask ML has been taken advantages of to predict the polymer–solvent miscibility. Active learning has also been used to make exploration of transition metal complexes. In the future work, more efforts can be made to investigate abundant types of materials assisted by AI to achieve the large-scale exploration and accelerated material discovery. c) In regard to the in-depth cognition of existing materials, the mapping of physical stimuli to a variety of perceptual features is in high demand. Detailed maps have led to a better understanding of visual and auditory coding. A principal odor map has also been proposed for olfactory perception. Accordingly, future research can be conducted focused on the revealing of the relationships between the physical stimuli and more diverse perceptual characteristics.

**Table 1:** Summary of the state-of-the-art AI systems empowered material science

Material system	Database	Method	Aim	Accuracy	Achievement	Other key features	Refs
Two-dimensional perovskites	by conducting high-throughput experiments	Subgroup discovery and support vector machine	To rapidly screening out materials with high synthesis feasibility	The trained ML model in good performance with an accuracy of 85%	To increase the success rate of the synthesis feasibility by a factor of four relative to traditional approaches	To be effective in a typical laboratory with limited experimental resources	[87]
PVDF-based nanogenerators	Experimental data for various TENG fabrication parameters	DTR, RF, and GBR	To predict performance	The GBR model exhibiting the highest R^2^ value of 0.9812 and 0.9370 for train and test	–	With real-time voltage output analysis	[96]
Sodium-ion batteries	DFT	SMOTEENN classifier	To predict the structural stability for materials discovery	With *R*^2^ value of 0.962 (Pristine)	To suggest optimal LTMO candidates with both the high energy density and electrochemical stability	–	[18]
High energy lithium-ion batteries	From experiments	LSTM	To develop high energy Ni-rich cathodes	Prediction accuracy of 80% (except for the stirring rate)	To make a prediction about the reaction conditions for synthesizing cathode precursors with specific morphologies	With the best cathode materials showing a high discharge capacity of 206 mAh g^−1^ at 0.1C and 83% capacity retention after 200 cycles	[97]
Passivation materials for perovskite solar cells	From a wealth of documented small molecules as passivation materials	SVM, NNM, RF, KNN, and NB	To justify dominant molecular traits and screen excellent passivation materials	SVM’s accuracy was the highest, exceeding 80%	To increase absolute efficiency values by over 2% with a champion efficiency of 25.41%	With limited available dataset	[98]
Crystalline porous materials	From the developed MatFormer	KCL	To make prediction of gas adsorption	To realize an 18% increase in *R*^2^	To realize a 20–35% decline of the mean absolute error compared to graph neural network CGCNN and ML models based on descriptors	To be universal for predicting the different physicochemical properties of diverse crystalline materials	[102]
Polymer–solvent	From experiments and quantum chemical calculations	DNN	to predict polymer–solvent miscibility	With the solubility classification accuracy of 0.857	To overcome the shortage of the quantitatively limited and qualitatively biased dataset was	To offer a highly generalized model for a wide range of polymer solution spaces	[108]
Oxides and phosphates	Historical data from the literature	Active learning	For the accelerated synthesis of novel materials	–	To realized 41 novel compounds from a set of 58 targets after continuous operating for over 17 days	To be integrated with robotics	[101]
Multicomponent oxides	Material phases and formation energies got from Materials Project	Thermodynamic strategy	To guide inorganic materials synthesis	–	224 reactions spanning 27 elements with 28 unique precursors could be accomplished by the robot under the operation of 1 human experimentalist	To be combined with robotic materials synthesis laboratory	[88]
Nanomaterial	From experiments	Quality-diversity algorithms	To explore and optimize nanomaterials	To realize a yield of up to 95%	To discover five categories of nanoparticles by only performing ca. 1000 experiments in three hierarchically linked chemical spaces	To be integrated with robot, and to transfer materials as seeds between cycles of exploration	[89]
Inorganic material	First-principles calculations	GNNs	For materials discovery	–	To improve the efficiency of materials discovery by an order of magnitude	2.2 million stable crystals have been found by GNoME models with respect to previous work	[51]
Odorants	To curate a reference dataset of ~ 5000 molecules	MPNN	To map molecular structure to odor perception	–	To outperform chemoinformatic models on several odor prediction tasks	To be contiguous, hierarchical, simple, and parseable	[53]
Battery	The Oxford Battery Degradation Dataset	DNN	To predict battery charging curve	To capture the charging curves accurately with an error of less than 16.9 mAh for 0.74 Ah batteries	To estimate the entire constant-current charging curves	To be adaptable to different batteries without much training effort and to be available with flexible input collected during daily charging	[180]
CO_2_ hydrogenation on supported cobalt catalysts	With the experimental, theoretical, and elemental parameters	symbolic-regression AI	To design materials for catalysis	–	To obtain a model well representing the experimental CH_3_OH selectivity	To identify key parameters as materials genes	[91]
Low-contact electrode for 2D semiconductor transistor	From the C2DB database	Autoencoding regularized adversarial neural network and a feature-adaptive variational active learning algorithm	To Screen electrode	With the mean square errors of 0.17 and 0.27 eV for the vertical and lateral Schottky barrier	To be trained with only 15% of the total data points	To be cost-effective and scalable	[157]
Solid-state electrolytes	Structure candidates based on ionic substitution to known crystal structures	With state-of-the-art ML models and traditional physics-based models	To realize screening in large scale and experimental validation	-	To quickly navigate through more than 32 million candidates and predict around half a million potentially stable materials	To combine ML with HPC	[99]
Metal oxides	From experiments	GA-adjusted ANN	To exploit gas-sensing descriptors	With *R*^2^ value of 0.92	-	-	[170]
Electrocatalysts for complex C–C coupling reaction networks	Iterative sampling	2D-3D ensemble ML	To reveals C–C electro coupling mechanism	–	To prove the ability of big dataset generated from ML to accelerate quantum chemical computations	With big dataset analysis	[171]
Biochar-based electrodes for supercapacitors	From recent articles	GBR	To clarify relationship between biochar preparation procedures and capacitance characteristics	with an *R*^2^ value of 0.93	–	–	[172]
Hierarchical porous carbon	From papers	ANN	To predict advanced potassium-ion batteries	With the MAE values of 8.830 and 2.390 for the capacity and ICE	To predict the relationship between different factors and the performance	–	[114]
Compounds for optoelectronic devices	–	Evolutionary search algorithms	To explore new compounds with structures similar to Haeckelites	–	To discover 13 new Haeckelite compounds		[52]

**Table 2:** Summary of some database used in materials science

Name	Contents	Sources and scale	Aim
The Materials Project	Data and associated analysis algorithms	With all known solid-state materials, thousands more unknown materials, over 30,000 stable materials and over 6,000 materials with computed elastic tensors	To provide open web-based access to computed information on known and predicted materials as well as powerful analysis tools to inspire and design novel materials
OQMD	DFT calculated thermodynamic and structural properties	With 1,317,811 materials	To search for material compositions, create phase diagrams, and visualize crystal structures
NOMAD	Data	Combining data from popular sources like the Materials Project, AFLOW, and OQMD	To extract and publish structured data with rich metadata
AFLOW (Automatic FLOW for Materials Discovery)	Data and calculated properties	Database of 3,929,948 material compounds with over 817,429,184 calculated properties, and growing	–
JARVIS (Joint Automated Repository for Various Integrated Simulations)	Data	Using classical force-field, density functional theory, machine learning, quantum computation calculations, and experiments	To automate materials discovery and optimization
Catalysis-Hub	Data and software	With thousands of reaction energies and barriers from density functional theory (DFT) calculations on surface systems	For computational catalysis research
ICSD	Database for completely determined inorganic crystal structures	Containing an almost exhaustive list of known inorganic crystal structures published since 1913, including their atomic coordinates	To find answers on questions in materials research
Crystallography Open Database (COD)	Data and software	Currently there are 527,197 entries in the COD	To provide open-access collection of crystal structures of organic, inorganic, metal–organic compounds and minerals, excluding biopolymers
NIST Materials Data Facility (MDF)	Data	> 650 Datasets, > 80 TB of Materials Data Published, and > 100 Data Sources Indexed	For publish, discover, and access materials datasets
PubChem	Data for chemical information	With 122MCompounds, 338MSubstances, 297MBioactivities, and 44MLiterature	To search chemicals, find chemical and physical properties, biological activities, safety and toxicity information, patents, literature citations
ZINC	Data	With over 230 million purchasable compounds in ready-to-dock, 3D formats. ZINC also contains over 750 million purchasable compounds you can search for analogs in under a minute	For virtual screening
PolyInfo	Data	The main data source is academic literature on polymers. With 19,227 homopolymers, 8,321copolymers, 2,788 polymer blends, 3,209 composites, 174,968 polymer sample, 552,427 property points, and 21,793 literature data	To provide various data required for polymeric material design

**Table 3:** Summary of various algorithms and models for different types of materials

Types of materials	Algorithms/Models	Aim	Experimental performance	Achievement	Refs
Catalysts	Large language models (LLMs) and genetic algorithms (GAs)	For the development of high-entropy alloy (HEA) catalysts	The optimal IrCuNiPdPt/C catalyst exhibits the record-low HER overpotentials at 10 and 100 mA cm^−2^, surpassing commercial Pt/C by 49% and 18%	To slash the discovery time from millennia to hours	[181]
Catalysis	Meta-learning model	For selectivity prediction in asymmetric catalysis	–	To demonstrate significant performance improvement over other popular ML methods, like random forests and graph neural networks	[182]
Polymers/ Hydrogels	Gaussian process (GP) and random forest regression (RFR)	For the design of high-performance adhesive hydrogels	To realize remarkable enhancement in adhesive strength, with a maximum value exceeding 1 MPa	To optimize hydrogel formulations from an initial dataset of 180 bioinspired hydrogels	[183]
Polymers	Feed-forward neural network	To accelerate the discovery of heat-resistant polysulfates	With a polysulfate exhibiting good thermal resilience and ultrahigh discharged energy density with over 90% efficiency at 200 °C	To provide the prediction of key proxy parameters and down selection of polymer candidates from a library of nearly 50,000 polysulfates	[184]
Catalysts	Crystal graph convolutional neural networks	To design a high-entropy intermetallic compound for catalyzing oxygen reduction reaction (ORR)	The catalyst with small particle size is successfully synthesized with ultrahigh mass activity and specific activity	To provide a high prediction accuracy with mean absolute errors of 0.003 for surface strain and 0.011 eV atom 1 for formation energy	[185]
Crystals	Graph networks	For improving the efficiency of materials discovery	Among the stable structures, 736 have already been independently experimentally realized	To enable the discovery of 2.2 million structures below the current convex hull	[51]
Catalysts	Regression models	To accelerate the discovery of catalysts	The screened irregular catalyst shows outstanding sulfur and moisture resistance and long-term stability (> 7000 h, *T*90 = 345 °C)	The high prediction accuracy could be realized with a small-size training set	[186]
Catalysts	Extra Trees model	For accelerated screening of single-atom anchored MXenes electrocatalyst	Experimental validation was achieved by 10 synthesized MXene-SACs	With an effective intrinsic descriptor to accelerate the high-throughput screening without additional computations	[187]
